# Running Together: How Sports Partners Keep You Running

**DOI:** 10.3389/fspor.2022.643150

**Published:** 2022-03-16

**Authors:** Rob Franken, Hidde Bekhuis, Jochem Tolsma

**Affiliations:** ^1^Department of Sociology, Radboud University, Nijmegen, Netherlands; ^2^Department of Orthopedagogics: Learning and Development, Radboud University, Nijmegen, Netherlands; ^3^Department of Sociology, University of Groningen, Groningen, Netherlands

**Keywords:** sports participation, social networks, sports partners, social comparison, running, motivation

## Abstract

We examined how recreational runners benefit from running with others to maintain a consistent training regimen over time. We used data from the ABS project (“Always Keep Active”). Our sample consisted of more than 800 individuals who had registered to participate in the 2019 edition of the 7K or 15K Seven Hills Run (Nijmegen, The Netherlands) for the first time. Taking advantage of this three-wave, individual-level panel data, we found that increases over time in the number of co-runners (of any ability level) are related to increases in the number of weekly running sessions. The probability of turning up at the Seven Hills Run was positively related to the number of equally or less competent co-runners, and to the number with whom respondents also discussed important matters on a frequent basis. Our recreational athletes differed in the extent to which they expressed social motivations to run. However, among these athletes, the positive impact of sports partners on sport outcomes did not depend on the importance of social motives. Our study demonstrates that social networks play an important role in maintaining a consistent training habit and in reaching set goals (i.e., participating in a race).

## Introduction

Running has become an increasingly popular way to become physically active and is ranked among the most popular sports in many countries. Every runner, from novice to experienced, has their own reasons for putting on their running shoes. They may train to become fitter (task involved) or to outperform others (ego involved; Roberts and Walker, 2020); they may be intrinsically motivated to run (e.g., because they enjoy it), extrinsically motivated (e.g., to impress friends), or may even run for no particular reason (Ryan and Deci, 2017). However, while many people take up running every year, it is similarly true that many give up soon. The real challenge seems to be maintaining a consistent training regimen for a longer period of time (Biddle and Mutrie, 2007).

Previous research indicates that all sports, even individualistic sports like running, are deeply socially structured (Scheerder et al., 2015). While it is possible to run alone and outside of any organization, many runners readily gather as members of formalized sports clubs, commercial gyms, or informal groups. They often adopt distinct social roles in these contexts, such as instructors, supporters, or competitors (Keegan et al., 2009), and off-track, obsessive running and rivalry are often exchanged for companionship and conversation (Jarvie et al., 2017). Runners are interconnected, and so is their running behavior. Therefore, if we want to understand why some runners keep going and others give up, we should not focus solely on individual factors (e.g., motivations) without concurrently addressing (changes in) the social context.

Growing evidence suggests that social networks may have profound implications for participation in sport. Several mechanisms that underlie the motivational role of social networks have been identified, such as social support (Sheridan et al., 2014), social facilitation (Evans et al., 2013), collective efficacy (Cohen et al., 2006), social identification (Stevens et al., 2019), and social comparison (Shakya et al., 2015). Traditionally, most studies on social influences on sports activity have simply focused on benefits of different types of relationships. For instance, Keresztes et al. (2008) measured social influence by asking respondents to what extent parents, siblings, classmates, and friends engage in sporting activities. Other studies have investigated how myriad social perceptions (e.g., social support, social identification) affect sports activity measures.^1^ Although several studies have demonstrated the interesting and varying significance of a multitude of social factors, research on how the act of *doing sports together* helps people to keep active remains unexplored.

In the present contribution, we focus on the social influence of co-runners on running persistence. Unlike previous studies that have focused simply on the benefits of different types of social ties, such as parents, partners, and coaches (Jetten et al., 2012), we investigate the social network formed by the people our respondents consider to be important sports partners and with whom they go running; we label this the “Core Sports Network” (CSN). Runners may form many sporting ties (e.g., in sports clubs or in online communities such as Strava), but the Core Sports Network (CSN) consists of strong sporting ties and reflects the inner circle of someone's personal sports network. Members of this network are expected to play an important role in the continuation of running activities.

The literature on motivation theories in sports and performance is vast and complex, and a thorough discussion of this is beyond the scope of the present contribution [see Roberts et al. (2018) for a review of the literature]. Most relevant for this study is the realization that running persistence is likely to be influenced by interactions of an individual, in combination with his/her specific set of motivations, within a larger social system in which that individual is embedded (Golden and Earp, 2012). The extent to which important co-runners affect a person's training habits may, in part, depend on that person's motivations for participating in sports. We will, therefore, assess the extent to which the impact of (changes in) the CSN on running is conditional on runners' motivations.

In sum, in this study, we will investigate how people's CSN impacts running regimens over time and assess the extent to which the social influence of the CSN depends on runners' motivations. While the relevance of both social influence (e.g., Fitzgerald et al., 2012) and motivation (Standage and Ryan, 2020) for sports participation have been established before, our study holds several innovative elements. First, we contribute to the literature because we integrate individualistic perspectives on sports participation (i.e., motivation) with broader social perspectives on sports participation (i.e., social networks). Furthermore, we go beyond earlier studies with a theoretical focus on a novel type of social network, namely, the CSN, thereby paying explicit attention to the motivational effect of running together and the impact of changes in that network. Moreover, our study, unlike the majority of related (longitudinal) studies in exercise literature, is not an intervention study but instead uses a large sample of adult runners who are followed for a relatively long period. Lastly, we do not focus on taking up sports (Kraaykamp et al., 2013) but on the continuation of running, a sport in which dropout rates are known to be high (Fokkema et al., 2019).

We will use the longitudinal panel dataset ABS [“Always Stay Active” (“*Altijd Blijven Sporten*”)]^2^ (Franken et al., 2020) to answer our research questions. This data set consists of a sample of 802 Dutch runners who registered for the 7K or 15K Seven Hills Run of 2019 but had not previously participated in this event. The Seven Hills Run (“de Zevenheuvelenloop”) is one of the largest road races in the Netherlands, taking place annually in mid-November. With the ABS data set, we are not only able to track changes in self-reported running frequency over time but also in the CSN as well as in other social aspects of the sporting environment.

## Theoretical Considerations

### Running Activity

In sports literature, exercise is quantified using the training variables frequency, duration, and intensity (e.g., Noakes, 2003). Novice runners are commonly advised to first increase training frequency to three times a week. Once a consistent training habit is built and/or an initial plateau is reached, runners may consider increasing their training load further by either increasing running frequency, workout duration, or workout intensity. In this article, we will focus on (changes in) running frequency. Although we acknowledge that running frequency may vary as a result of training periodization, and as decline in running frequency does not necessarily signal running dropout, we will interpret consistency in running frequency or increases in running frequency as a persistent running habit.

Moreover, as the second indicator of a persistent running habit, we will investigate whether runners who registered for the Seven Hills Run also crossed the (start and) finish line. Goals are universal in sports: nearly all athletes set goals on a frequent basis to structure training and motivate performance (Munroe-Chandler et al., 2004). Setting a running goal like participating in the Seven Hills Run provides direction to athletic pursuits and lays the foundation for continuous sports participation (Williams, 2013). Runners who did not start the Seven Hill Run may have set too ambitious a goal or simply abandoned it and are assumed to be at greater risk of losing a persistent running habit. Conversely, attending the event and attaining the goal may elevate psychological wellbeing and motivation, thereby advancing personal growth and development (Smith et al., 2007).

### The Core Sports Network

A body of evidence shows that having a strong network helps enable the pursuit of sports over time (e.g., Biddle and Mutrie, 2007; Keegan et al., 2011; Fitzgerald et al., 2012). Earlier studies have examined various sources of social influence: significant others, such as family members and friends, physicians or work colleagues, fitness instructors, and other co-exercisers. The network we focus on in the present contribution is the CSN, which is made up of key people with whom we run. Two major mechanisms through which significant co-runners can motivate an individual's long-term continuation of sports are related to social support and social comparison (Zhang et al., 2016).

Social support is one of the most widely used and studied strategies for encouraging healthy behaviors in social networks (Berkman et al., 2000; Eaker, 2005). It can be understood as: [the] “aid and assistance exchanged through social relationships and interpersonal transactions” (Heaney and Israel, 2008, p. 191). Social support, thus, refers to supportive actions of the social environment. It encompasses various categories, such as instrumental support (e.g., providing material assistance), appraisal support (e.g., offering companionship), informational support (e.g., providing advice), and emotional support (e.g., encouragement). Support derived from interpersonal relationships in a sporting context has been identified as an important resource for athletes (Sheridan et al., 2014).

An alternative approach to promoting sports and health behaviors utilizes social comparison *via* social relations (Buunk et al., 2013). Social comparison refers to individuals' innate drive to generate stable and accurate appraisals of themselves by searching out comparative standards. These social comparisons are used to understand one's position in relation to others, to evaluate this position, and to act upon this evaluation (Festinger, 1954). Doing sports together allows people to compare themselves with their co-exercisers. Training habits of the people we consider important co-runners may serve as running goals to strive for, resulting in improved activity levels (Shakya et al., 2015).

The greater the number of significant people with whom individuals run, the more likely they are to receive social support (Seeman and Berkman, 1988; Martí et al., 2017). Similarly, a larger network of key co-runners is expected to provide a positive basis for social comparison (Sterling, 2013). We, thus, expect the size of the CSN to positively affect the frequency with which an individual runs and that runners with larger CSNs are more likely to show up at the Seven Hills Run for which they registered (hypothesis 1b). We expect hypothesis 1 to hold even if we take into account the influence of sporting ties outside the CSN [e.g., ties with(in) informal running groups and online running communities].

A lot of work has been devoted to social comparisons in health and sports specifically (e.g., Bardel et al., 2010; Evans et al., 2013; Park and Park, 2017). Social comparison can be either downward or upward, depending on whether individuals compare themselves to those who perform worse or better than them (Festinger, 1954). The variants of social comparison yield different underlying motivational processes, with both different benefits and drawbacks. Upward social comparison is argued to motivate self-improvement, the aim being to approximate better-performing alters (Lockwood and Kunda, 1997). Recent experimental evidence suggests that when people are surrounded by peers who exercise more than themselves, they become motivated to increase their activities, which eventually results in a “social ratchet effect” for the entire group (Zhang et al., 2016). Following this line of thought, we expect that better-performing key co-runners in particular (i.e., who are more competent) will spur an individual to increase their running frequency (hypothesis 2a). Moreover, in particular, we expect that these better-performing alters increase an individual's chance of turning up at the race (hypothesis 2b). Having a reference group of high-performing co-runners may ignite the desire for activity, resulting in increased likelihood of attending a running event.

Whereas previously, also adverse effects of upward comparison were suggested—upward comparison may threaten one's self-view (Corcoran et al., 2011) and may result in a loss of motivation (Mollee and Klein, 2016)—a review of studies testing upward comparison effects on self-evaluation, self-esteem and affect concluded that upward comparison is not in conflict with the desire for positive self-regard, but rather serves it indirectly through self-improvement and sometimes directly by self-enhancement (Collins, 1996, 2000). Conversely, downward comparison may positively affect sports persistence by boosting one's self-view, esteem, and wellbeing (Wills, 1981). However, downward comparison may result in relatively low goals, since it does not challenge individuals to self-improve to minimize discrepancy with their sports partners.

### Interplay Between Social Networks and Social Sports Motivations

In our contribution, we are not so much interested in how different types of motivation are related to sport outcomes - in line with earlier research, we expect that task-orientated and intrinsically motivated runners will be especially able to maintain a consistent running regimen (Standage and Ryan, 2020). Rather, we are interested in the extent to which the impact of the CSN on running persistence varies according to athletes' motivations. More precisely, we focus on whether the presumed positive impact of co-runners is greater among athletes who express strong social motivations to run.

According to the social production function (SPF) theory (Lindenberg, 1996), people strive for two universal goals: physical and social wellbeing. Running together with the CSN is multifunctional in that it produces both physical and social wellbeing (Fujimoto et al., 2018). Doing sports together with important sports partners serves to promote physical wellbeing through the stimulus and activity provided, and social wellbeing through social aspects, such as expressing and refining social identity (Barber, Stone, Hunt and Eccles, 2005). We have argued that two major social functions of running together are related to social support and social comparison. We expect that, especially for athletes who express strong social motivations to run and, more precisely, who run to maintain friendships and/or to enhance peer status, these functions of running together are important. Hence, we hypothesize that the presumed positive relationships between, on the one hand, CSN size and, on the other hand, running frequency and attendance rates, are stronger for runners who express strong social motivations to run (hypotheses 3a and 3b).

## Methods

### Data

Individual-level longitudinal panel data from the ABS project [“Always Stay Active” (in Dutch: “*Altijd Blijven Sporten*”)] were used to test our hypotheses. The ABS data set includes data on 802 unique adult respondents^3^ and information was collected on, for example, reasons why recreational athletes run, their self-reported running frequencies, important people with whom they run, and (other) social aspects of the sports environment.

To recruit respondents for this data-collection project, invitation emails (see Supplementary Material) were sent to runners who enrolled for the Seven Hills Run for the first time and who, during the enrollment process, ticked a box giving permission to be sent invitations to participate in scientific research. The survey was administered *via* the online survey tool LimeSurvey (Schmitz, 2020) hosted on university servers. All respondents who filled out the first online survey (September 2019) were asked whether they were willing to participate in subsequent survey tasks. These respondents were invited to participate in the second (end of October 2019) and third survey rounds (end of April 2020). Respondents received 7.5-euro and 10-euro gift cards after completion of the questionnaire of waves 1 and 3, respectively. The anonymized data set has been deposited in the online repository DANS EASY. For more information on data collection and response rates, we refer to the codebook (Franken et al., 2020).

### Dependent Variables: Running Frequency and Attendance

In the first round of survey (September 2019), respondents were asked about their average *running frequency* over the past 12 months and over the past month (in times per week). In the second round, in the week leading up to the race (October 2019), they were again asked about their running frequency over the past month. In the third round (April 2020), they were asked about their running frequency in the month of February and March as measures for running frequency prior to and during the coronavirus disease-2019 (COVID-19) outbreak. This resulted in five observations of running frequency per respondent. For analyses, we used only the first four observations, because doing sports together during the pandemic may have been influenced by opinions about restrictive measures that were in place back then. Frequencies per week were converted to times per year. Extreme values (more than 364 days a year) were excluded from the analyses (~3%).^4^ This resulted in values of running frequency ranging from 6 to 364 times a year.

The official website of the Seven Hills Run was used to obtain finish times. Runners with a finish time were given a score 1 on our second (dichotomous) dependent variable *show up*, and runners without a finish time 0. DNF (did not finish) is not registered on the website of the Seven Hills Run, but DNF rates are negligible (< 0.5%).

### Core Sports Network

To tap into runners' CSN, we asked the respondents to name the most important people they had been running with over the last 12 months (wave 1) and over the last month (wave 2). The respondents could name up to 5 core sports partners. In the third round of survey the respondents were asked if they still run with the people they reported in the previous rounds.

#### Network Size

Based on this information, we constructed a time-varying variable reflecting the size of the CSN, ranging from 0 to 5. Network studies generally show diminishing marginal returns for additional network partners (e.g., Marinazzo et al., 2012; Semrau and Werner, 2014). A preliminary analysis, using a method previously applied by Semrau and Werner (2014) and described in detail by Wooldridge (2015), also showed this to be the case in our study. We, therefore, used the square root term as our measure of CSN size.

#### Co-Runner Competence

Once the most important sports partners had been named, the remaining questions (“name interpreters”) focused on the names mentioned. The respondents were asked how competent at running their sports partners were, with values ranging from “much worse than me” to “much better than me” (1–5). We counted the *number of more competent co-runners* and the *number of co-runners who were no better* (as a reference group). Both variables are entered in our model as square root versions. In additional analyses, we also investigated the impact of less competent co-runners (downward comparison). To this end, we constructed separate variables for the number of better, equal, and worse co-runners (see Robustness paragraph).

#### Co-Runner Tie Strength

The people making up our CSN were argued to constitute strong sporting ties. One of the name interpreter questions for each sports partner referred to the extent to which they were also people with whom the respondents discussed important matters (cf. Marsden, 1987). A core discussion network question is generally perceived to “delineate the inner core of one's personal network, which consists of the most intimate ties” (Mollenhorst et al., 2014, p. 68). Core discussants are theorized to be a major source of social support (Small et al., 2015), and co-runners with whom an individual discusses important matters are, thus, expected to be especially important in the continuation of running activities. For additional analyses (see Robustness paragraph), we counted the number of CSN members with whom an individual discussed important matters frequently (“strong ties”) and the number with whom they did not (“weak ties”).^5^

#### Other Social Aspects of Sporting Environment

To take into account possible other running ties outside the CSN, the respondents were asked in wave 1 and wave 3 about the setting in which they run. We constructed four dichotomous variables indicating whether the runners are *member of a sports club* (1 = yes, 0 = no), *member of a commercial gym* (1 = yes, 0 = no), run in *informal groups* (e.g., organized by family or friends) (1 = yes, 0 = no), or run *alone* (1 = yes, 0 = no).

In the first round of survey, we also asked the respondents whether they used digital applications (e.g., Strava, RunKeeper). Based on this information, we constructed a time-invariant dichotomous variable *activity in online sports networks* (1 = yes, 0 = no).

### Motivations

A Dutch version of Sport Motivation Scale (SMS-6; Mallett et al., 2007) was included in our data set. The stem “Why do you go running?” was followed by different phrases tied to different types of motivation. The scale was traditionally designed to measure different motivational regulations as distinguished in self-determination theory (SDT; Ryan and Deci, 2017; amotivated, external, introjected, identified, integrated, and intrinsic). Response scales were 7-point Likert scales ranging from “does not correspond at all” to “corresponds exactly.” We used the SMS-6 rather than other popular sport motivation scales [e.g., the SMS-II of Pelletier et al. (2013) and the BRSQ of Lonsdale et al. (2008)], as it better taps into social motivations to run (see the online Supplementary Material for a comparison of the scales). More precisely, it contains two motives that clearly reflect social motivation: (a) running for companionship (“I run because it is one of the best ways to maintain good relationships with my friends”) and (b) peer status (“I run to show others how good I am at my sport”). These motives correspond theoretically well to the mechanisms of social support and comparison, respectively. We take as our measure for social motivation the standardized weighted sum score for the social motivation dimension, which considers the unique contribution of both manifest items to the latent variable they are measuring (DiStefano et al., 2009; see Appendix A for more details on the inter-factor correlations of SMS-6 we used).

Additionally, recognizing the need to take into account the multidimensionality of motivation (Ullrich-French and Cox, 2009), we did not focus solely on social motivations but instead classified our runners according to different motivational profiles guided by SDT, and investigated whether the impact of the CSN varied across athletes exhibiting different motivational profiles (see Robustness paragraph).

### Controls

Control variables can be grouped into two clusters. The first cluster considers temporal resources available to respondents, as decisions regarding sports are highly dependent on people's free time. Family composition, parenthood, and employment status were taken into account. Dummy variables were made for different types of family composition: (1) living together with a partner and children living at home, (2) living together without children living at home, (3) living alone with children living at home, (4) living alone without children living at home. Dummy variables reflecting the respondents' occupational setting were included: (1) full-time employment, (2) part-time employment, (3) student, and (4) others.

The second cluster includes time-stable demographic variables: sex, age, and educational level. Education was recoded into four categories for which we constructed dummy variables: (1) “primary,” (2) “lower secondary,” (3) “higher secondary,” and (4) “tertiary.” Lastly, we included the distance the respondents registered for (i.e., 7 or 15 km).

### Between-Within Model

The ABS is a panel study of 802 unique respondents, of whom 549 also participated in wave 2 and 569 also participated in wave 3. We have 2,596 unique observations. To exploit the panel structure of our data and include all the observations, we estimated a so-called between-within model, also known as the hybrid model (Allison, 2009). The formal model in its basic form is:


(1) $$y_{ti}=\beta_{0}+\beta_{1}(x_{ti}-{\bar{x}}_{i})+\beta_{2}{\bar{x}}_{i}+\beta_{3}z_{i}+u_{0i}+e_{ti},$$

with *y*_*ti*_ being the running frequency of respondent *i* at time-point *t*. *x*_*ti*_ is a (time-varying) indicator of respondent *i* at time-point *t* (e.g., CSN size). ${\bar{x}}_{i}$ is the mean score of actor *i* of this indicator across time-points. *z*_*i*_ is a time-stable indicator of respondent *i* (e.g., social motivation). *u*_0*i*_ and *e*_*ti*_ are normally distributed error terms, with *u*_0*i*_ representing individual-level random intercepts (deviations of the mean score of *y* of individual *i* from the grand mean, β_0_) and *e*_*ti*_ representing observation-specific deviations. The estimated parameter β_1_ represents the within-person effect of *x* on *y*, and these parameters are identical to parameters we would obtain from standard fixed effects approaches (i.e., including a dummy variable for every respondent in the regression model). β_2_ and β_3_ represent the between-person effects of *x* and *z* on *y*. In models with a parameter referring to the (cross-level) interaction between *x* and *z*, we included a random slope in our model [i.e., we replaced the original parameter β_1_ of the formula above with a fixed ($\left(\beta_{1}^{\prime }\right)$ and random part (*u*_1*i*_)], which was allowed to covary with the random intercept. The models were estimated with the R-package “lme4” (Bates et al., 2015).

## Results

### Descriptive Statistics

**Figure 1:**
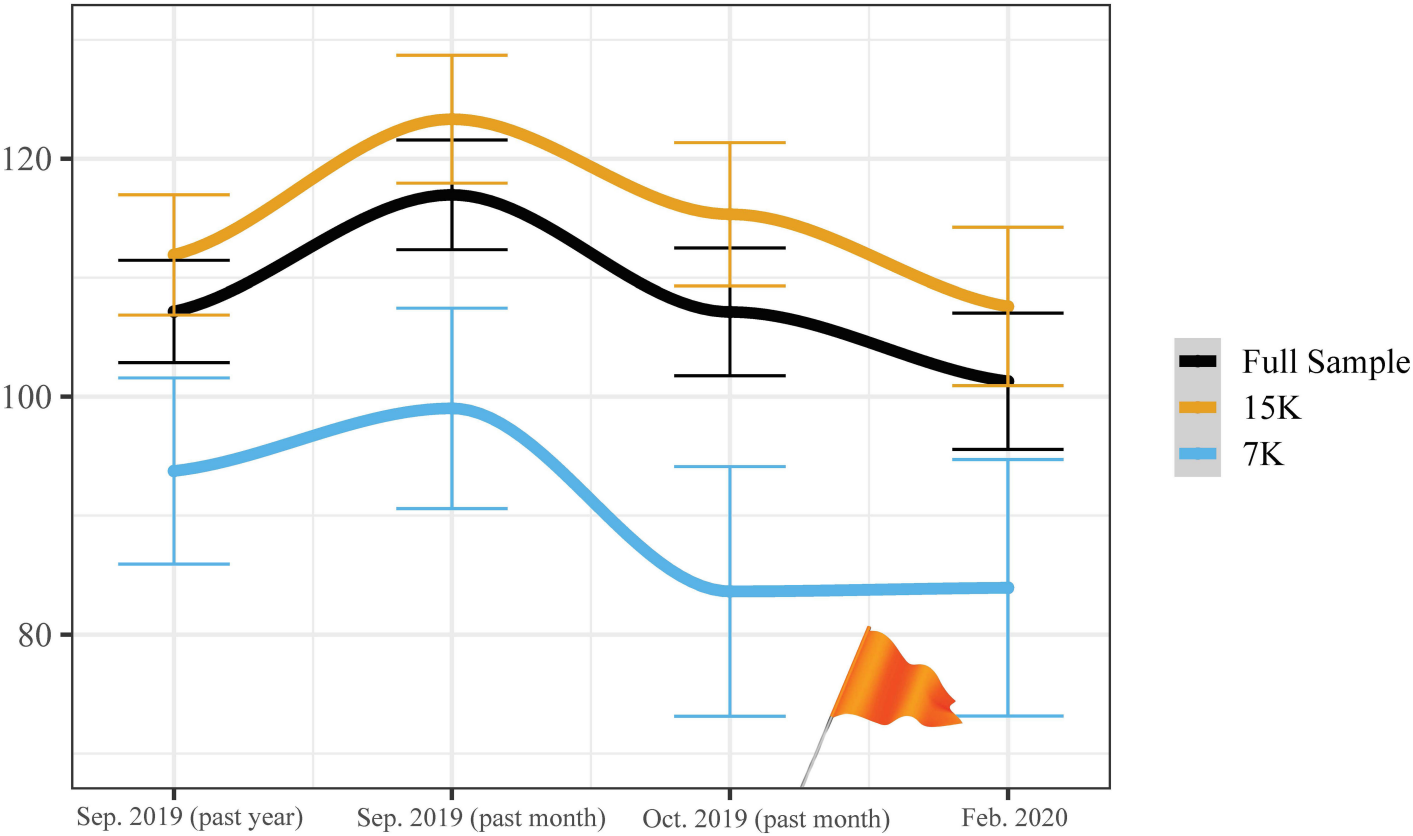
Development of running frequency (converted to times per year). Horizontal lines depict error bars representing the standard error of the mean. Orange flag represents the Seven Hills Run (November 14 and 15).

Descriptive statistics of the study variables can be found in the online Supplementary Material. On average, the respondents reported running between 100 and 117 times per year over the four observations. Figure 1 illustrates the development of grand mean yearly running frequency over time, disaggregated by the Seven Hills Run (15 km) and the Seven Hills Night (7 km). It demonstrates that there is a clear pattern in the development of the mean running frequency, which may imply the existence of both seasonal effects and training periodization.

Moreover, 20% of the respondents we included in the analyses of *show up* did not start the race. Preliminary analyses demonstrated that running frequency and show up are strongly interrelated yet distinct theoretical indicators for running persistence (see Appendix B).

In other preliminary analyses, we assessed how the CSN was formed and who it was made up of (see Appendix B). It seemed that the CSN is very diverse. This echoes our argument that to map an individual's strong sporting ties, it is more fruitful to rely on their own assessment of who is important than on a taxonomic approach (i.e., focusing on different social roles, such as partners and friends).

### Impact of CSN on Running Frequency

Tables 1, 2 present the results of the between-within models. In Table 1 the impact of the size of the CSN is described; in Table 2, the impact of the number of better co-runners and number of co-runners who are no better is assessed separately.

**Table 1:** Between-within effects of Core Sports Network (CSN) size on running frequency, panel analysis.

	**Model 1**		**Model 2**		**Model 3**	
	**b**	**SE**	**b**	**SE**	**b**	**SE**
(Intercept)	117.12^***^	12.77	86.80^***^	15.79	87.45^***^	15.79
**Within-effects**						
CSN size	4.40^*^	2.13	3.95^~^	2.15	3.38	2.64
Sports club			15.61^*^	6.90	18.34^*^	7.14
Commercial gym			6.99	7.34	7.38	7.50
Informalgroup			−1.70	5.14	−2.82	5.15
**Between-effects**						
CSN size	6.85^*^	3.14	0.44	3.30	0.48	3.30
Sports club			28.96^***^	5.21	28.89^***^	5.22
Commercial gym			17.70^*^	7.72	18.05^*^	7.75
Informalgroup			6.20	6.19	6.62	6.24
Online sports network			6.55	4.67	6.36	4.68
Socialmotivation	6.66^**^	2.02	4.59^*^	2.03	4.49^*^	2.05
**Cross-level interaction**						
CSN size * socialmotivation					0.93	2.42
Individuals (*n*)/observations (*N*)	647/2,229		647/2,229		647/2,229	
Marginal *R*^2^/conditional *R*^2^	0.083/0.567		0.121/0.570		0.122/0.607	

~ *p < 0.1*.

* *p < 0.05*.

** *p < 0.01*.

*** *p < 0.001 (two-tailed)*.

*Only explanatory variables of interest are shown, excluding controls and time fixed-effects. Also, only fixed intercepts (β_0_) and slopes ($\beta_{1}^{{}^{\prime }}$) are shown, and not the random parts (u_0i_ and u_1i_). All coefficients not shown are available upon request*.

**Table 2:** Between-within effects of number of more and equally/less competent co-runners, separately.

	**Model 1**		**Model 2**		**Model 3**	
	**b**	**SE**	**b**	**SE**	**b**	**SE**
(Intercept)	109.07^***^	17.71	110.19^***^	17.72	109.84^***^	17.62
**Within-effects**						
CSN size	4.99	3.37				
More competent co-runners			7.83^~^	4.25	6.60	4.89
Equally/less competent co-runners			−1.06	3.78	−3.36	4.19
**Between-effects**						
CSN size	−0.20	3.46				
More competent co-runners			−4.83	4.37	−5.14	4.35
Equally/less competent co-runners			4.94	4.02	5.38	4.01
Sports club	24.58^***^	5.52	24.89^***^	5.53	24.45^***^	5.52
Commercial gym	14.74^~^	7.63	15.32^*^	7.64	14.91^~^	7.65
Informalgroup	4.80	6.17	5.32	6.17	4.61	6.17
Online sports network	7.82	5.24	8.31	5.24	8.02	5.24
Social motivation	3.27	2.26	2.99	2.26	2.96	2.77
**Cross-level interaction**						
Higher^*^ social motivation					4.04	4.71
Equal/lower^*^ social motivation					0.66	3.88
Individuals (*n*) /observations (*N*)	642/1,117		642/1,117		642/1,117	
Marginal *R*^2^/conditional *R*^2^	0.113/0.560		0.116/0.560		0.114/0.657	

~ *p < 0.1*.

* *p < 0.05*.

** p < 0.01.

*** *p < 0.001 (two-tailed) Only explanatory variables of interest are shown, excluding controls and time fixed-effects. Also, only fixed intercepts (β_0_) and slopes ($\beta_{1}^{{}^{\prime }}$) are shown, and not the random parts (u_0i_ and u_1i_). No within-effects of sports setting (e.g., sports club) are estimated, as this variable was invariant between waves 1 and 2. All coefficients not shown are available upon request*.

In model 1 (Table 1), we include as a predictor variable the size of the CSN (at the between- and within-level) and social motivation measured at time 1. Model 1 also includes time fixed effects and our control variables. The effect of the size of the CSN on running frequency is positive at the between-level (b = 6.85, SE = 3.14) and the within-level (b = 4.4, SE = 2.13). Our findings, thus, suggest that runners who have more important co-runners run more frequently, and that increases in the size of the CSN drive up running frequency.

To rigorously assess the impact of the CSN, in model 2 we controlled for other possible (changes in) relevant sports environments (clubs, groups, gyms, and online communities). In this model, the between-effect of CSN size is small and not significant. However, the effect at the within-level remains positive (b = 3.95, SE = 2.15, Table 1, model 2) and, given our directional hypothesis, significant at α <0.05 (*p* = 0.034, one-tailed),^6^ lending support for hypothesis 1a.

We predicted that better co-runners, in particular, will influence an individual's running frequency (hypothesis 2a). In testing this hypothesis, we only used data from waves 1 and 2 (hence the lower N). First, we re-estimated our previous model including the size of the CSN with this restricted sample. The effect of the size of the CSN is positive but does not reach significance. In model 2 (Table 2), we replaced CSN size with our measures for the number of better co-runners and co-runners who are no better (at the between- and within-level). The within-effect of the number of better sports partners is positive (b = 7.83, SE = 4.25, Table 2, model 2) and significant (*p* = 0.033, one-tailed). Sports partners of equal or lower competence compared to the respondent (the reference group) do not affect running frequency (b = −1.06, SE=3.78). This indicates that the magnitude of the effect of the CSN is driven by co-runners who are more competent, lending support for hypothesis 2a.

**Figure 2:**
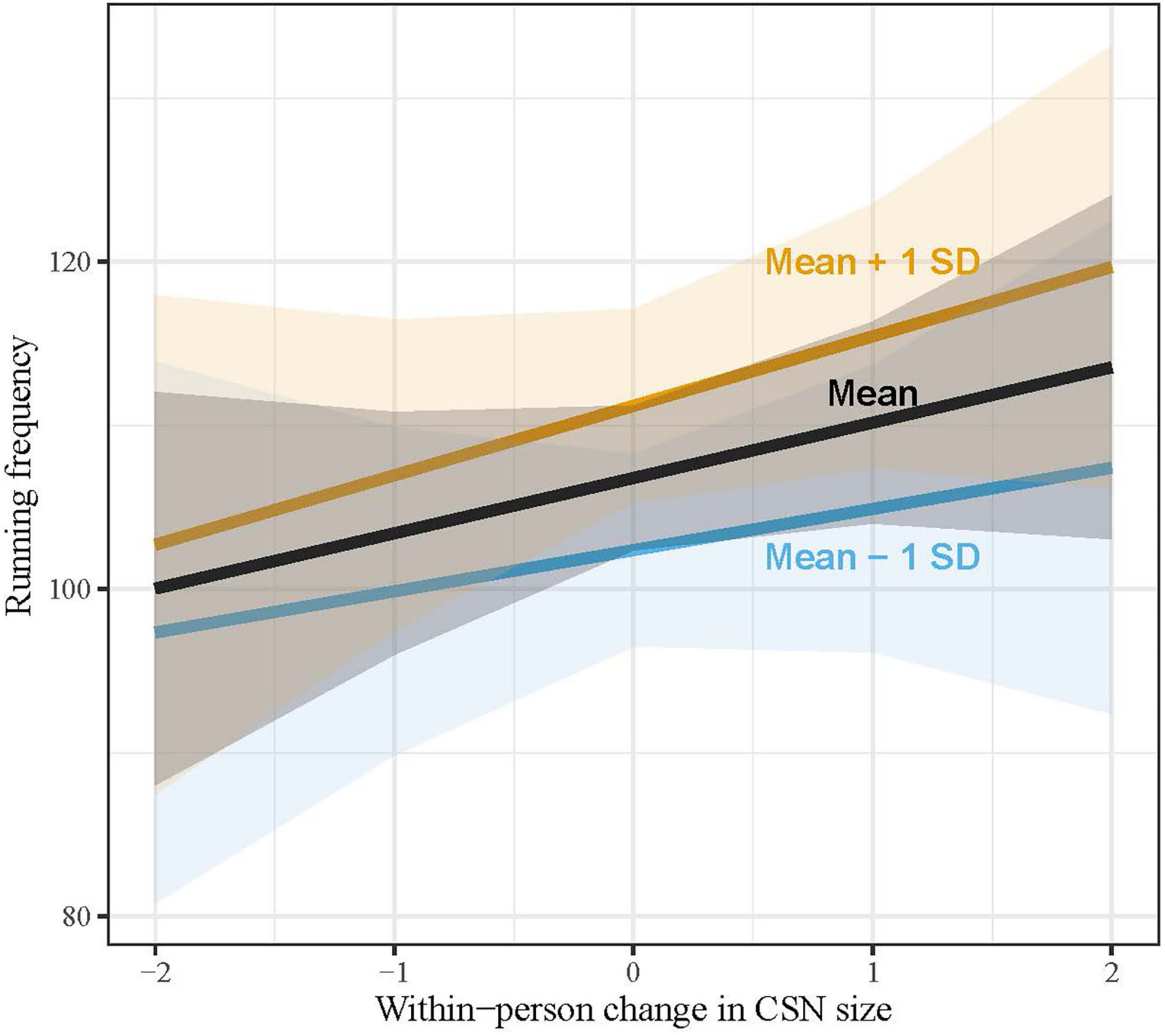
Impact of changes in Core Sports Network (CSN) size on running frequency, moderated by social motivation. Lines reflect predicted values of running frequency for runners with average social motivation, below average (-1 SD), and above average (+1 SD) based only on fixed effects estimates (Table 1, model 2), with shaded areas reflecting 90% confidence intervals. X-axis labels refer to the within-individual changes in CSN size [i.e., deviations from person-specific mean $\left(x_{ti}-{\bar{x}}_{i}\right)$].

In hypothesis 3a, we formulated the expectation that, especially for athletes who express strong social motivation, CSN size affects running. Our results indicate that runners who acknowledge social motives to a greater extent run more frequently (b = 6.66, SE = 3.14, Table 1, model 2). In model 3 (Table 1), we introduced the (cross-level) interaction between, on the one hand, size of the CSN at the within-level, and, on the other hand, social motivation at the between-level. The cross-level interaction was not significant, indicating that the impact of changes in the size of the CSN on running frequency is much the same for athletes with varying degrees of social motivation. Figure 2 illustrates this interaction: the slopes do not differ significantly. We must, therefore, reject hypothesis 3a. In model 3 (Table 2), we introduced the cross-level interaction between, on the one hand, the number of better co-runners and number of co-runners who are no better (separately), and, on the other hand, social motivation. As with our previous model, we observe that the impact of these co-runners did not vary across runners with different levels of social motivation.

The focus of this contribution is on how changes in the constellation of the CSN affect an individual's running frequency. We controlled for other possible (changes in) relevant sports environments. With respect to these other social contexts, we find that becoming a member of a sports club is related to increase in running frequency (b = 15.61, SE = 6.9, Table 1, model 2).

To illustrate the effect of the CSN, let us consider that a respondent named 1, 2, and 3 co-runners in the consecutive waves (${\bar{x}}_{i}=\frac{\sqrt{1}+\sqrt{2}+\sqrt{3}}{3}$ = 1.38).^7^ Based on this information, combined with the parameter estimates for the fixed intercepts (β_0_ = 86.8), the within-effect of CSN size (β_1_ = 3.95) and the between-effect (β_2_ = 0.44), the estimated running frequency at baseline (W1) is ~86 times per year (86.8 + 3.95^*^($\sqrt{1}$ – 1.38) + 0.44^*^1.38). The estimated running frequency in W3 is ~89 times per year (86.8 + 3.95^*^($\sqrt{3}$ – 1.38) + 0.44^*^1.38). In other words, an increase in the CSN by two co-runners over the course of 8 months, with all else equal, drives up yearly running frequency by 3%. Even though the within-effects of the CSN are statistically significant, their absolute impact on running frequency seems only marginal. However, if we compare these effects to other effects in our model, they appear to be rather substantial. As an example, the within-effect of CSN size is nearly equal to the effect of our social motivation measure at the between-level, which indicates that the impact on running frequency of a 1-alter increase in the CSN approximates that of a 1-SD increase in social motivation. Moreover, the within-effect of the number of better co-runners is approximately one-third of the between-effect of sports clubs, which is the largest effect in our models.

### Impact of CSN on Showing Up

To assess the impact of the CSN on showing up at the Seven Hills Race, we estimated logistic regression models with the size of the CSN in W2 (weeks before the race) on the probability of appearing at the start.

In model 1, we included as predictor variables the size of the CSN, social motivation, and control variables. The results are presented in Table 3. As expected, the more CSN members that someone names as part of their event preparation, the more likely they are to appear at the start (OR = 1.64, Table 3, model 1). In model 2, we controlled for other social contexts in which a person may have running ties, but this does not change the effect of CSN size.

**Table 3:** Lagged logistic regression effects on showing up at a running event.

	**Model 1**		**Model 2**		**Model 3**		**Model 4**	
	**OR**	**CI**	**OR**	**CI**	**OR**	**CI**	**OR**	**CI**
(Intercept)	1.23	0.27 – 5.95	1.33	0.28 – 6.72	1.35	0.28 – 6.86	1.25	0.26 – 6.35
CSN size	1.64^***^	1.23 – 2.22	1.64^***^	1.22 – 2.25			1.63^**^	1.21 – 2.23
More competent co-runners					1.25	0.84 – 1.91		
Equally/less competent co-runners					1.79^**^	1.20 – 2.74		
Sports club			0.85	0.50 – 1.49	0.87	0.51 – 1.52	0.84	0.49 – 1.46
Commercial gym			0.75	0.37 – 1.63	0.77	0.38 – 1.67	0.76	0.37 – 1.66
Informalgroup			1.45	0.77 – 2.90	1.50	0.79 – 3.01	1.45	0.77 – 2.90
Online sports network			0.90	0.54 – 1.48	0.93	0.55 – 1.53	0.91	0.54 – 1.50
Socialmotivation	1.12	0.91 – 1.40	1.13	0.90 – 1.42	1.12	0.90 – 1.42	1.05	0.80 – 1.38
CSN size ^*^ socialmotivation							1.16	0.86 – 1.58
Observations	649		649		649		649	
*R*^2^ Tjur	0.042		0.046		0.048		0.047	

* *p < 0.05*.

** *p < 0.01*.

*** *p < 0.001 (two-tailed). The dependent variable show up was coded so that 0 = did not show up and 1 = did show up. OR = odds ratio; CI = 95 % confidence interval; significance is based on log(odds). Only explanatory variables of interest are shown, excluding controls. All coefficients not shown are available upon request*.

In Model 3, we make a distinction between co-runners who are better and no better than the respondent. It seems that running with the latter, in particular, increases the likelihood of appearing at the start (OR = 1.79). The number of better co-runners also has a positive effect, albeit not significant.

**Figure 3:**
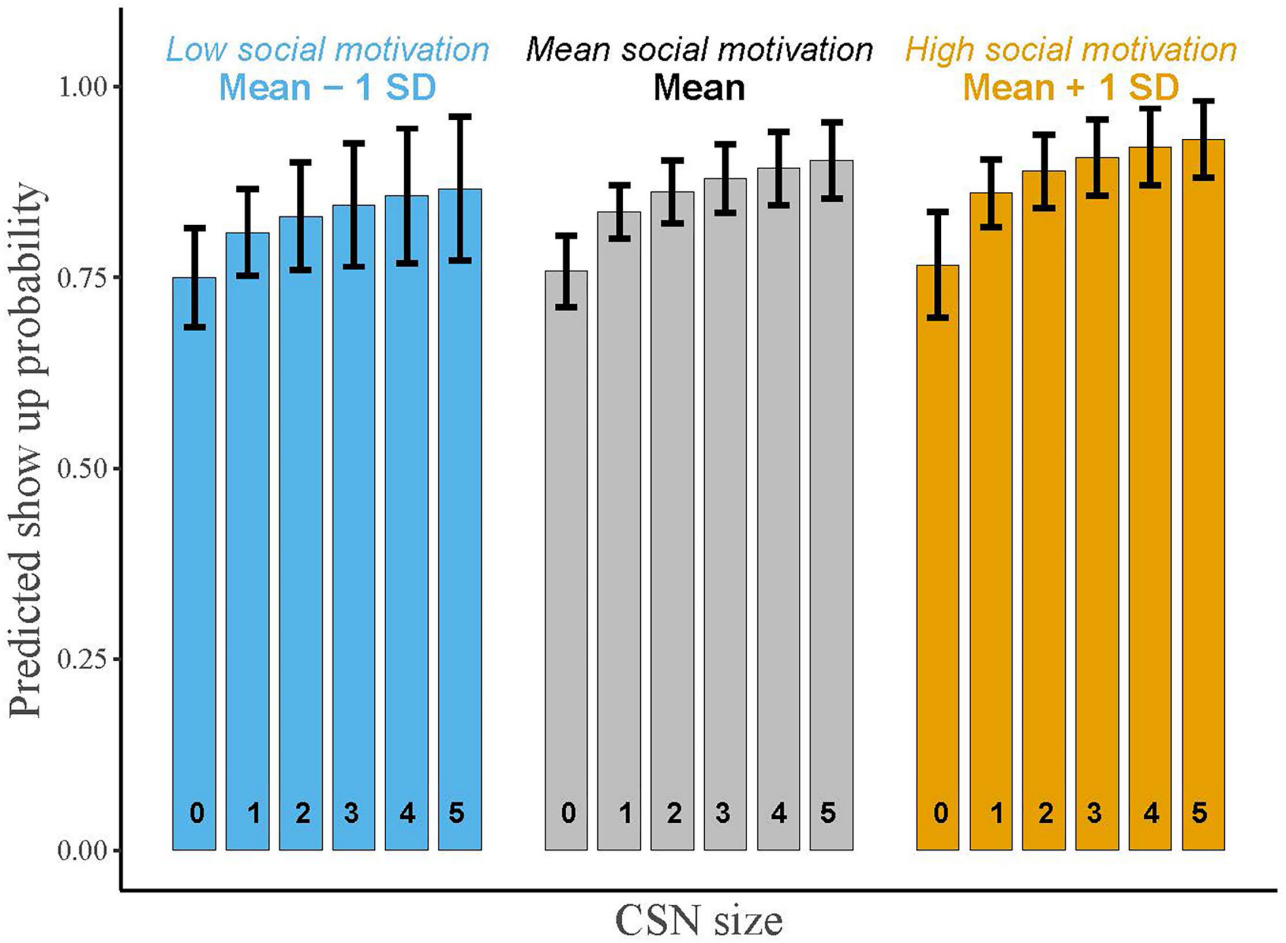
Predicted probabilities of showing up at a running event, moderated by social motivation. Bars reflect predicted probabilities of showing up at the Seven Hills Run, conditional on social motivation (mean −1 SD, mean, mean +1 SD), with error bars reflecting 95% confidence intervals. X-axis labels refer to the number of important co-runners in W2. Probabilities are based on estimates in Table 3 (model 3).

Furthermore, we see that athletes exhibiting higher social motivation are not more likely to show up. In model 4, we included the interaction between the size of the CSN and social motivation. The impact of size of the CSN on the likelihood of showing up was not considerably nor significantly different for athletes with different levels of social motivation (see Figure 3). The impact of the number of either better or equal/worse co-runners was also not conditional on social motivation (not shown).

### Robustness Checks

#### Different Network Measures

As a first robustness check, we re-estimated our between-within models with separate variables for the number of more, equally, and less competent co-runners (see Appendix C). Now, both the numbers of more competent co-runners and less competent co-runners have a positive effect on running frequency at the within-level, but the number of sports partners of equal competence has a negative within-effect. Additionally, we did not find interaction effects between these terms and social motivation. Therefore, contrary to our hypothesis, both upward and downward comparisons seem to drive running frequency, even when other comparisons are controlled for. On the other hand, lateral comparison (i.e., with equally competent co-runners) seems to be ineffective or even counter-effective.

Second, we replaced our network measures with the number of “strong ties” and “weak ties” (see Appendix C). We observe that (increases in) these strong ties were not necessarily important for driving up running frequency. However, in our logistic models predicting whether or not runners would show up at the Seven Hills Run, it appeared that it was the number of strong ties in particular that affected attendance rates. In addition, based on mediation analysis, we cautiously conclude that sports partners who are not more competent may improve the likelihood of attending a running event, in part because they are often strong (supportive) ties.

#### Different Operationalizations of Motivation

Athletes can maintain simultaneously many different reasons to do sports. Indeed, the items that constitute our social motivation measure are (moderately to strongly) positively correlated to other items of the SMS-6 referring to extrinsic or intrinsic types of motivation, and negatively to items tapping into amotivation (see Appendix A). We re-estimated our models by controlling for standardized weighted sum scores of the other subdimensions of the SMS-6. Still, we do not find corroborative evidence that the effects of the CSN are conditional on athletes' social motivation (see Appendix C).

Additionally, we classified our runners into different motivational profiles by latent profile analysis (cf. Emm-Collison et al., 2020; see Appendix C). We identified three such profiles based on statistical criteria and guided by theoretical considerations following self-determination theory (SDT). Not surprisingly, high-motivation runners run more frequently, but the impact of the CSN did not vary across runners with different motivational profiles.

## Discussion and Conclusion

It is an urgent societal problem that physically inactive lifestyles are becoming ubiquitous. As sports represent a key means of keeping physically active while offering multiple other benefits, it is important to understand how individuals can be motivated to pursue sports over time. In this study, we sought to investigate how (change in) the constellation of the Core Sports Network is related to running activity. We followed two major arguments made in the literature on social networks and health, i.e., that social ties can improve a person's participation in sports through supportive actions, and that the process of social comparison may motivate individuals to self-improve, the aim being to approximate better-performing sports partners.

Our main finding is that changes in the constellation of the CSN over time drive changes in running frequency. When the number of significant running partners increases, the number of running sessions also increases. We expected that when runners can name more key co-runners, they receive more social support to run and will have a positive basis for social comparison. Even though we did not explicitly address these mechanisms empirically, we did investigate what kind of co-runners were important for attaining a persistent running habit. Co-runners with whom a person can self-compare seemed particularly important for motivating running frequency, which echoes the previous experimental evidence of Zhang et al. (2016), while supportive co-runners seemed important for attending the Seven Hills Run, which is in line with previous research on the influence of social support on health goal attainment (e.g., VonDras and Madey, 2004).

To rigorously assess the impact of important co-runners, we controlled for other possible changes in relevant sports environments. We observed that becoming a member of a sports club drove up running frequency, and that this partly mediated the effect of the size of the CSN. While sports clubs may increase social exposure to potential new important co-runners, an increase in the number of people with whom you go running may also increase one's likelihood of joining a running club. However, the question remains what the relative importance is of both directions of causality.

We expected the CSN to be more important for runners with social motivational reasons to run (i.e., for companionship and/or for peer status). However, the impact of co-runners was not conditional on runners' degree of social motivation, even when controlling for other types of motivation. Given the complex interplay of different types of motivation to do sports, we also explored whether the impact of the CSN differed across runners with different motivational profiles. However, we did not find any empirical evidence for this. Therefore, while motivations may impact running frequency directly, we found robust evidence that network effects are not conditional on the type of motivations that athletes express, regardless of the motivational operationalization we use.

Our results underline the importance of focusing on social networks in sports promotion programs. By demonstrating that sports partners play an important role in keeping people active, even in an individualistic sports like running and even for athletes exhibiting high-motivation profiles, this study echoes previous calls for network interventions (e.g., Maturo and Cunningham, 2013). Whereas previous physical activity interventions focusing on behavior change have only had a minimal positive impact (Metcalf et al., 2012), focusing on social networks may be more effective (e.g., Gesell et al., 2012). Social networks are, moreover, prominent targets for cost-effective interventions (Maher et al., 2015), and may have a more sustainable impact on a person's sporting behavior. Besides, an important observation for intervention designers and practitioners is that effective influence on persistent training is generated through social comparisons in particular. Whereas social support from significant others is generally believed to contribute to the adoption and maintenance of sports activities, both in the literature (e.g., Wendel-Vos et al., 2007) and in interventions (e.g., Eime et al., 2008; Ooms et al., 2013), we showed that among athletes who are already running and planning on taking part in a race, comparison processes are especially important for increasing training frequency. It seems plausible that social networks are effective sources of social support in relation to taking up sports, but that social comparison processes orient people who are already active to remain so over time.

We employed name interpreter questions to map the characteristics of important co-runners, but these questions also entail limitations on our study. Most notably, we assessed how competent co-runners were *relative* to our respondents. While this allowed us to standardize the gap between the respondents and their co-runners, it complicates the interpretation of network changes. More precisely, an increase in the number of important co-runners who are no better than the respondents is ambiguous, in that it may imply (a) that a respondent expanded their network with less competent co-runners and (b) that the number of less competent co-runners increased as a result of a respondent increasing their running frequency and competence. In other words, measuring co-runner competence in relative terms in a longitudinal design raises the potential concern that we overestimated the (positive) effects of downward comparison. Conversely, our design might underestimate the effects of upward comparison if a respondent decreases their training, and, subsequently, their self-assessed running competency: previously named co-runners become more competent in relative terms, even if they do not increase their running frequency and/or competence.

Another limitation is that we tracked athletes over the course of ~1 year. We recognize that sustained sporting activity lasts longer than this. Not being an experimental or intervention study but focusing on a large sample of runners and their self-reported CSNs, is a strength of this study but also raises “reflection” issues (Manski, 1993). This means that the process of social influence, the confounding by shared exogenous social environments of social network members, and endogenous social selection cannot be isolated from each other (Shalizi and Thomas, 2011).

Based on these limitations, we encourage future research to measure alter characteristics (e.g., competence and frequency) not only in relative terms, but also in absolute terms. Also, including more alter characteristics (name interpreters) is necessary for understanding who exactly aids in reaching fitness goals: supportive co-runners, co-participants, fellow club members, or others? Moreover, future scholars may separate the effects of social influence and selection by collecting longitudinal data with more waves and using statistical methods that simultaneously model both causal pathways, such as the random intercept cross-lagged panel model (RI-CLPM; Hamaker et al., 2015). Future research should investigate the explanatory mechanisms of social support and comparison directly, for example, by measuring psychological variables, such as perceived social support and comparison. We demonstrated convincingly that network effects are not conditional on individual motivations, but we invite future researchers to replicate our study using a different measure of social motivational orientations (e.g., the SMOSS; Allen, 2005).

We suspect that changes in CSN have even more pronounced effects on running frequency than we were able to show in this article. First, our available data on running frequency were not very detailed: we used ordinal categories to measure weekly and/or monthly running frequency, and we recoded this to the number of times someone went running per year in total, settling for the fact that this could slightly differ from their actual running frequency. In addition, the use of these original ordinal data raises the concern that we were unable to expose changes in running frequency that stay within the range of one answer category. Second, we should be aware that we have a specific sample consisting of adult runners who enrolled in a big running event and who were, therefore, likely, or at least more likely than beginners, to have already achieved a consistent training routine. The specificity of our sample in conjunction with a possible measurement error in the dependent variable may have resulted in underestimation of changes in running frequency and, more importantly, less power to detect effects of changes in the CSN. Third, even though the CSN reflects the inner circle of someone's personal sports network, naturally there are several other relevant networks that may promote sports participation. Besides, while we controlled for several of these potential networks (e.g., online networks, such as Strava), we were unable to analyze the network effects at play there.

Despite these limitations, we have made several contributions to the literature. We focused on a novel type of social network, the Core Sports Network, which we mapped using a name generator. Combining social network theories with psychological motivation theories, we integrated the individualistic perspective on motivation with a broader social perspective on social networks. Unlike the majority of (intervention) studies in exercise literature, we used a large sample of adult runners, and we focused not on taking up a sport but on running persistence. We clearly demonstrated that runners' social sporting network influences their training frequency and probability of showing up at a race. Whereas social networks may provide effective impetus for taking up sports, social comparison seems especially important when it comes to maintaining sports activity. This offers a promising avenue for future interventions aimed at keeping people active.

## Data Availability Statement

The datasets presented in this study can be found in online repositories. The names of the repository/repositories and accession number(s) can be found at: DANS (https://doi.org/10.17026/dans-zd5-bp24).

## Ethics Statement

Ethical review and approval was not required for the study on human participants in accordance with the local legislation and institutional requirements. The patients/participants provided their written informed consent to participate in this study.

## Author Contributions

All authors contributed to the conception and design of the research and the data collection. RF performed the analyses. The manuscript was written by RF with support and supervision from HB and JT. All authors contributed to the article and approved the submitted version.

## Funding

This study was supported by ZonMW Sportinnovator (grant no. 538001037) and NWO TRIAL (grant no. 24001475).

## Conflict of Interest

The authors declare that the research was conducted in the absence of any commercial or financial relationships that could be construed as a potential conflict of interest.

## Publisher's Note

All claims expressed in this article are solely those of the authors and do not necessarily represent those of their affiliated organizations, or those of the publisher, the editors and the reviewers. Any product that may be evaluated in this article, or claim that may be made by its manufacturer, is not guaranteed or endorsed by the publisher.

## Footnotes

^1^See Sheridan et al. (2014) and Chung et al. (2017) for reviews on social influences on sports activity.

^2^Funded by the Netherlands' Organization for Health Research and Healthcare Innovation (ZonMw).

^3^Apart from two respondents—who were 16 and 17—all respondents were aged 18-plus.

^4^The self-reported running frequencies of these respondents were physiologically impossible. Hence, we interpreted these values as reflecting measurement error.

^5^We asked respondents how often they had discussed important matters with their respective CSN members over the last 12 months, with response categories ranging from “almost every day” to “never” (1–7). We considered the people with whom individuals discussed important matters more than once a month to be “strong ties”; others were considered “weak ties”.

^6^We divide the two-tailed *p*-values from Table 1 by 2.

^7^We calculated the range in CSN size for each respondent as the difference between the highest CSN size and the lowest CSN size reported in the successive waves. Sixty-five percent of respondents has a CSN size range of 2 or more.

## Supplementary Material

Supplementary Datasheet 1 Supplementary material.

Supplementary Datasheet 2 Appendix A.

Supplementary Datasheet 3 Appendix B.

Supplementary Datasheet 4 Appendix C.
