# Supplementary material for: Running Together: How Sports Partners Keep You Running
Source: Front Sports Act Living. 2022 Mar 16;4:643150. doi: 10.3389/fspor.2022.643150 (PMC8966768; doi:10.3389/fspor.2022.643150)
Supplement: Supplementary Datasheet 1 — Supplementary material. [file Data_Sheet_1.docx]

Supplementary Material

**Table 1.** Descriptive statistics of time-varying study variables, disaggregated by observation

|  | Wave 1 | | | | Wave 2 | | Wave 3 | |
| --- | --- | --- | --- | --- | --- | --- | --- | --- |
|  | Time-point 1 (N=642) | | Time-point 2 (N=630) | | Time-point 3 (N=475) | | Time-point 4 (N=470) | |
|  | Mean | Std. dev. | Mean | Std. dev. | Mean | Std. dev. | Mean | Std. dev. |
|  |  |  |  |  |  |  |  |  |
|  |  |  |  |  |  |  |  |  |
| Running frequency (6-364) | 107.16 | 55.42 | 116.96 | 59.08 | 107.12 | 60.37 | 101.32 | 63.42 |
| CSN size (0-5) | 1.71 | 1.62 | 1.70 | 1.62 | 1.17 | 1.37 | 1.32 | 1.47 |
| Nr. of more skilled co-runners | 0.72 | 0.95 |  |  | 0.56 | 0.91 |  |  |
| Nr. of equally/less skilled co-runners | 0.99 | 1.19 |  |  | 0.61 | 0.87 |  |  |
| Sport setting (ref. = not) |  |  |  |  |  |  |  |  |
| Sports club | 0.20 |  | 0.20 |  | 0.21 |  | 0.23 |  |
| Commercial gym | 0.08 |  | 0.08 |  | 0.08 |  | 0.05 |  |
| Informal group | 0.15 |  | 0.15 |  | 0.13 |  | 0.15 |  |
| Alone, unorganized | 0.79 |  | 0.80 |  | 0.78 |  | 0.81 |  |
| Amotivated regulation^a^ | 1.37 | 0.70 | 1.38 | 0.71 | 1.35 | 0.67 | 1.36 | 0.73 |
| External regulation | 2.24 | 1.18 | 2.24 | 1.17 | 2.26 | 1.19 | 2.25 | 1.21 |
| Introjected regulation | 6.00 | 1.71 | 6.00 | 1.71 | 6.01 | 1.70 | 6.01 | 1.74 |
| Identified regulation | 3.86 | 1.44 | 3.87 | 1.44 | 3.89 | 1.45 | 3.84 | 1.46 |
| Integrated regulation | 4.82 | 1.62 | 4.81 | 1.62 | 4.92 | 1.59 | 4.88 | 1.63 |
| Intrinsic motivation | 4.44 | 1.75 | 4.44 | 1.75 | 4.49 | 1.78 | 4.52 | 1.78 |
| Social motivation | 2.12 | 1.13 | 2.12 | 1.13 | 2.16 | 1.13 | 2.17 | 1.16 |
| *Note*: statistics are based on the respondents that have valid responses on the dependent variable in the respective observations. ^a^ Statistics for the motivational regulations are based on the (unstandardized) weighted sum scores for the SMS-6 subdimensions. | | | | | | | | |

**Table 2.** Descriptive statistics of time-constant study variables, disaggregated by observation

|  | Wave 1 | | | | Wave 2 | | Wave 3 | |
| --- | --- | --- | --- | --- | --- | --- | --- | --- |
|  | Time-point 1 (N=642) | | Time-pont 2 (N=630) | | Time-point 3 (N=475) | | Time-point 4 (N=470) | |
|  | Mean | Std. dev. | Mean | Std. dev. | Mean | Std. dev. | Mean | Std. dev. |
|  |  |  |  |  |  |  |  |  |
| Show up (ref.=no) | 0.80 |  | 0.80 |  | 0.83 |  | 0.82 |  |
| Online network (ref.=no) | 0.79 |  | 0.79 |  | 0.83 |  | 0.81 |  |
| Motivational profiles (ref.=no) |  |  |  |  |  |  |  |  |
| Amotivated | 0.22 |  | 0.23 |  | 0.22 |  | 0.21 |  |
| Low motivation | 0.40 |  | 0.43 |  | 0.42 |  | 0.43 |  |
| High motivation | 0.38 |  | 0.34 |  | 0.36 |  | 0.36 |  |
| Gender (0=female, 1=male) | 0.49 |  | 0.49 |  | 0.49 |  | 0.49 |  |
| Age (16-81) | 41.08 | 11.30 | 41.09 | 11.29 | 41.34 | 11.62 | 41.25 | 11.62 |
| Education (ref. = not) |  |  |  |  |  |  |  |  |
| Primary education | 0.00 |  | 0.00 |  | 0.00 |  | 0.00 |  |
| Lower secondary | 0.03 |  | 0.03 |  | 0.03 |  | 0.03 |  |
| Higher secondary | 0.19 |  | 0.19 |  | 0.18 |  | 0.19 |  |
| Tertiary | 0.78 |  | 0.78 |  | 0.79 |  | 0.78 |  |
| Family composition (ref. = not) |  |  |  |  |  |  |  |  |
| Together with children | 0.43 |  | 0.43 |  | 0.41 |  | 0.41 |  |
| Together without children | 0.31 |  | 0.31 |  | 0.31 |  | 0.33 |  |
| Alone with children | 0.07 |  | 0.07 |  | 0.07 |  | 0.07 |  |
| Alone without children | 0.19 |  | 0.19 |  | 0.20 |  | 0.20 |  |
| Occupational setting (ref. = not) |  |  |  |  |  |  |  |  |
| Full time employment | 0.63 |  | 0.63 |  | 0.62 |  | 0.62 |  |
| Part time employment | 0.26 |  | 0.26 |  | 0.26 |  | 0.26 |  |
| Studying | 0.05 |  | 0.05 |  | 0.05 |  | 0.05 |  |
| Other | 0.06 |  | 0.06 |  | 0.07 |  | 0.07 |  |
| Run (0=7km, 1=15km) | 0.74 |  | 0.74 |  | 0.74 |  | 0.73 |  |
| *Note*: statistics are based on the respondents that have valid responses on the dependent variable in the respective observations. | | | | | | | | |

|  | **SMS-II (2013)** | **BRSQ (2007)** | **SMS-6 (2007)** |
| --- | --- | --- | --- |
|  | Stem: I practice my sport ... | Stem: I participate in my sport … | Stem: Why do you practice sport? |
|  | Anchors: 1 = *does not correspond at all*, 4 = *corresponds moderately*, 7 = *corresponds exactly* | Anchors: 1 = *Not at all true*, 4 = *Somewhat true*, 7 = *Very True* | Anchors: 1 = *does not correspond at all*, 4 = *corresponds moderately*, 7 = *corresponds exactly* |
|  | I used to have good reasons for doing sports, but now I am asking myself if should continue. | But I wonder what's the point.. | I don’t know anymore; I have the impression of being incapable of succeeding in this sport. |
| *Amotivation* | So that others will praise me for what I do. | But I question why I continue. | I don’t know if I want to continue to invest my time and effort as much in my sport anymore. |
|  | It is not clear to me anymore; I don't really think my place is in sport. | But the reasons why are not clear to me anymore. | It is not clear to me anymore; I don’t really think my place is in sport |
|  |  | But I question why I am putting myself through this. | I don’t seem to be enjoying my sport as much as I previously did |
|  | Because people I care about would be upset with me if I didn't. | Because if I don't other people will not be pleased with me. | Because it allows me to be well regarded by people that I know |
| *External* | Because I think others would disapprove of me if I did not. | Because I feel pressure from other people to play. | For the prestige of being an athlete |
|  | Because people around me reward me when I do. | Because people push me to play. | For the material and/or social benefits of being an athlete |
|  |  | In order to satisfy people who want me to play. | ***To show others how good I am at my sport*** |
|  | Because I would feel bad about myself if I did not take the time to do it. | Because I would feel ashamed if I quit. | Because it is absolutely necessary to do sports if one wants to be in shape |
| *Introjected* | Because I feel better about myself when I do. | Because I would feel like a failure if I quit. | Because I must do sports to feel good about myself |
|  | Because I would not feel worthwhile if I did not. | Because I feel obligated to continue. | Because I would feel bad if I was not taking time to do it |
|  |  | Because I would feel guilty if I quit. | Because I must do sports regularly |
|  | Because I have chosen this sport as a way to develop myself. | Because the benefits of sport are important to me. | Because it is a good way to learn lots of things which could be useful to me in other areas of my life |
| *Identified* | Because I found it is a good way to develop aspects of myself that I value. | Because it teaches me self-discipline | Because it is one of the best ways I have chosen to develop other aspects of my life |
|  | Because it is one of the best ways I have chosen to develop other aspects of myself. | Because I value the benefits of my sport. | ***Because it is one of the best ways to maintain good relationships with my friends*** |
|  |  | Because it is a good way to learn things which could be useful to me in my life. | Because training hard will improve my performance |
|  | Because practicing sports reflects the essence of whom I am. | Because it's a part of who I am. | Because it's part of the way in which I’ve chosen to live my life |
| *Integrated* | Because participating in sport is an integral part of my life. | Because it’s an opportunity to just be who I am. | Because it is an extension of me |
|  | Because through sport, I am living in line with my deepest principles. | Because what I do in sport is an expression of who I am. | Because participation in my sport is consistent with my deepest principles |
|  |  | Because it allows me to live in a way that is true to my values. | Because participation in my sport is an integral part of my life |
|  | Because it gives me pleasure to learn more about my sport. | Because I enjoy it. | For the excitement I feel when I am really involved in the activity |
| *Intrinsic* | Because I find it enjoyable to discover new performance strategies. | Because I like it. | Because I feel a lot of personal satisfaction while mastering certain difficult training techniques |
|  | Because it is very interesting to learn how I can improve. | Because it's fun. | For the satisfaction I experience while I am perfecting my abilities |
|  |  | Because I find it pleasurable. | For the pleasure of discovering new performance strategies |

Table 3. Comparing sport motivation scales

*Note*: items making up our social motivation measurement are in red.

**Project invitation emails**

Down below is the invitation email (translated into English) we sent to runners who enrolled for the Seven Hills Run for the first time and who, during the enrollment process of the event, ticked a box whereby they gave permission to receive invitations to participate in scientific research.

Dear {FIRSTNAME} {LASTNAME},

Great that you are participating in the Seven Hills Run or Seven Hills Night for the first time this year!

What were your motivations to enroll? How do you stay motivated to train and appear at the start in November?

Radboud University conducts research into motivation in athletes. We use this knowledge to get people exercising and keep them active. In this way, we try to contribute to a healthier society. You can help with that! Are you taking part in our Seven Hills research project? To participate, click the link down below.

{SURVEY URL}

During the road towards the Seven Hills, we would like to ask you a few questions about your motivations and the associated peaks and valleys. We would appreciate your participation! You participation is of course completely voluntary. However, it is very important for our research that you take part. After all, your opinions and experiences matter. To thank you for participating in our research, you will receive a voucher from *Runnersworld* of 7.50 Euro.

The first questionnaire is the longest. It will take about 15-20 minutes to fill out.

We have received your email address with your permission from the Seven Hills Run Foundation. Radboud University Nijmegen guarantees that your answers will only be used for scientific purposes. Of course, we adhere to the General Data Protection Regulation ([see here](https://autoriteitpersoonsgegevens.nl/nl/onderwerpen/algemene-informatie-avg/algemene-informatie-avg)) and we comply with our professional code ([see here](https://www.nsv-sociologie.nl/?page_id=17)).

We would like to thank you in advance for participating in our research. If you have any questions, please email or call us.

With sporty greetings,

Dr. Hidde Bekhuis
Dr. Jochem Tolsma
