# Supplementary material for: Running Together: How Sports Partners Keep You Running
Source: Front Sports Act Living. 2022 Mar 16;4:643150. doi: 10.3389/fspor.2022.643150 (PMC8966768; doi:10.3389/fspor.2022.643150)
Supplement: Supplementary Datasheet 2 — Appendix A. [file Data_Sheet_2.docx]

# Appendix A: factor structure and inter-factor correlations of the SMS-6

Confirmatory factor analysis [CFA; performed using the R-package ‘Lavaan’ (Rosseel, 2012)] yielded adequate model fit to the SMS-6 data (χ^2^ = 1123.633, df = 237, p<0.001; CFI = 0.850; RMSEA = 0.074; SRMR = 0.059). See Figure 1 for the confirmatory structure of the model. All items loaded onto their expected factors significantly. The items making up our social motivation measure – “… to show others how good I am at my sport” (orange) and “…because it is one of the best ways to maintain good relationships with my friends” (blue) – were originally designed to measure two distinct motivational regulations in the framework of SDT, i.e., external and identified regulation respectively. Results of the CFA revealed that these items had the poorest loading on the latent variables they were supposed to measure (see Table 1a). We estimated an additional factor model with these respective items loading onto a seventh factor which we label ‘social motivation’ (see Table 1b). This model yielded a slightly better fit than the initial CFA (χ^2^ = 1037.08, df = 231, p<0.001; CFI = 0.864; RMSEA = 0.072; SRMR = 0.056). We formally compared the model fit of both models through the Vuong test for non-nested models (Merkle et al., 2016; Vuong, 1989) using the R-package ‘nonnest2’ (Merkle and You 2018). The test of distinguishability based on the observed data was statistically significant (ω^2^ = 0.14, p<0.001) and the non-nested likelihood ratio test indicated that the model with the additional social motivation factor provided a better fit than the initial model ($z$ = -4.40, p<0.001).


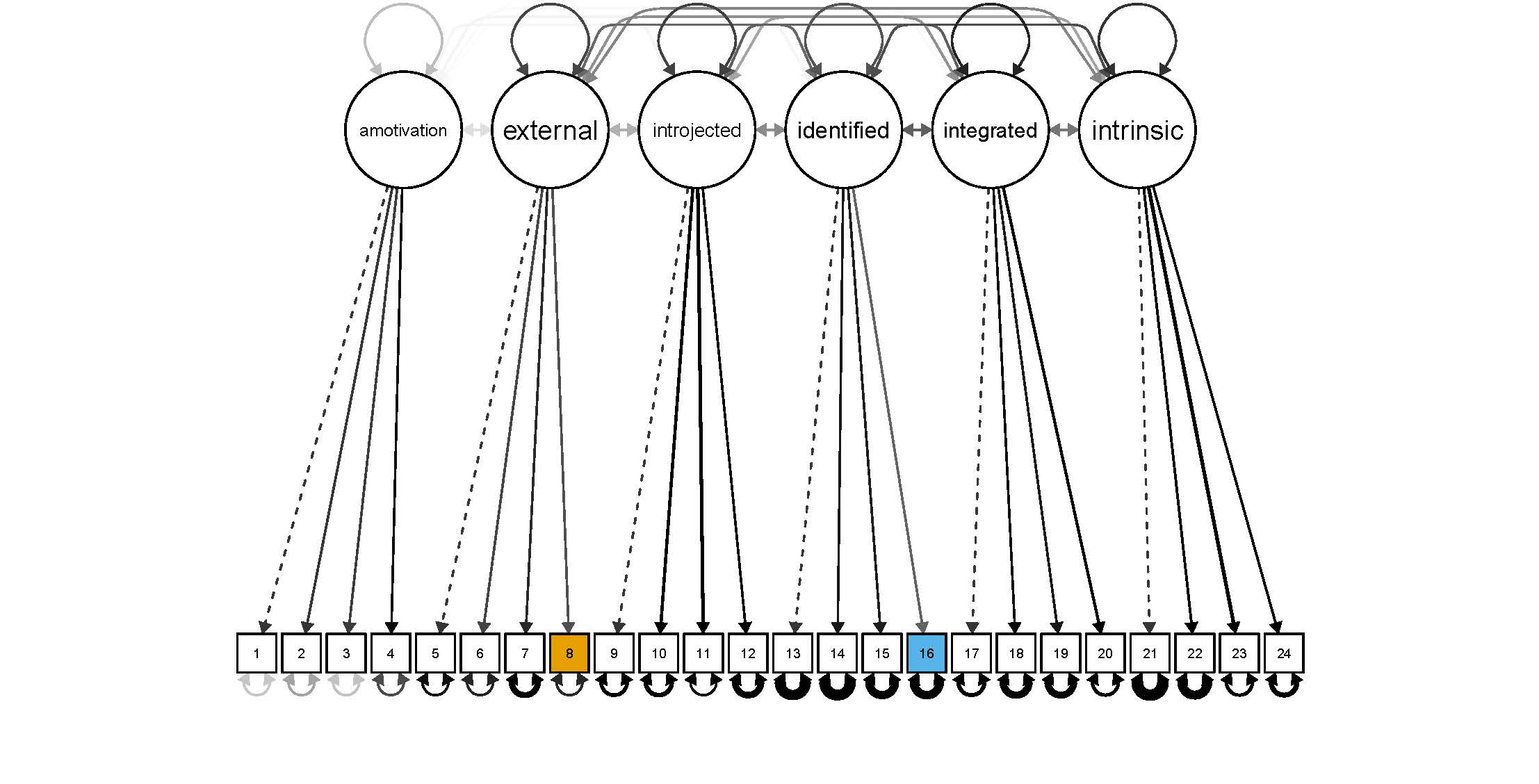

**Figure 1.** Confirmatory structure of the SMS-6 data based on SDT  *Notes*: Variables are represented as nodes, with square nodes indicating manifest variables and circular nodes indicating latent variables. Manifest variable 8 (orange) is “… because it is one of the best ways to maintain good relationships with my friends”; manifest variable 16 (blue) is “… to show others how good I am at my sport”. Relations between variables are represented by unidirectional and bidirectional edges, representing regression equations and (co)variances, respectively. Fixed parameters (i.c. scaled to 1) are visualized with dashed lines. All factor loadings and variances (i.e. loops) are significant at *α*<0.001 (see supplementary material). Fades are based on the size of the parameter estimates (larger estimates are darker than smaller estimates) and path width is based on the error estimates (larger errors are thicker than smaller errors). The diagram was generated using the R-package ‘semPlot’ (Epskamp, 2015).

**Table 1a.** Factor loadings of SMS-6 data

| **Latent Factor** | **Indicator** | **B** | **SE** | **Z** | **Beta** | **sig** |
| --- | --- | --- | --- | --- | --- | --- |
| amotivation | W1_M1_5 | 1.000 | 0.000 |  | 0.733 |  |
| amotivation | W1_M2_4 | 1.004 | 0.073 | 13.700 | 0.645 | *** |
| amotivation | W1_M3_1 | 0.969 | 0.067 | 14.470 | 0.705 | *** |
| amotivation | W1_M3_6 | 1.198 | 0.095 | 12.650 | 0.583 | *** |
| external | W1_M1_4 | 1.000 | 0.000 |  | 0.660 |  |
| external | W1_M2_3 | 0.932 | 0.069 | 13.483 | 0.652 | *** |
| external | W1_M3_3 | 1.067 | 0.082 | 12.951 | 0.618 | *** |
| external | W1_M3_8 | 0.873 | 0.066 | 13.135 | 0.629 | *** |
| introjected | W1_M1_7 | 1.000 | 0.000 |  | 0.633 |  |
| introjected | W1_M2_2 | 1.342 | 0.090 | 14.877 | 0.729 | *** |
| introjected | W1_M2_8 | 1.388 | 0.090 | 15.400 | 0.771 | *** |
| introjected | W1_M3_7 | 1.260 | 0.089 | 14.158 | 0.680 | *** |
| identified | W1_M1_3 | 1.000 | 0.000 |  | 0.527 |  |
| identified | W1_M1_8 | 1.146 | 0.098 | 11.740 | 0.575 | *** |
| identified | W1_M3_4 | 1.125 | 0.094 | 12.028 | 0.597 | *** |
| identified | W1_M2_7 | 0.778 | 0.078 | 9.959 | 0.455 | *** |
| integrated | W1_M1_2 | 1.000 | 0.000 |  | 0.658 |  |
| integrated | W1_M2_1 | 1.210 | 0.081 | 14.916 | 0.683 | *** |
| integrated | W1_M2_5 | 1.150 | 0.078 | 14.660 | 0.668 | *** |
| integrated | W1_M3_5 | 1.308 | 0.081 | 16.163 | 0.759 | *** |
| intrinsic | W1_M1_1 | 1.000 | 0.000 |  | 0.550 |  |
| intrinsic | W1_M1_6 | 1.278 | 0.103 | 12.448 | 0.669 | *** |
| intrinsic | W1_M2_6 | 1.400 | 0.104 | 13.412 | 0.769 | *** |
| intrinsic | W1_M3_2 | 1.254 | 0.097 | 12.898 | 0.712 | *** |
| *Note*: *p<0.05; **p<0.01; *** p<0.001 | | | | | | |

| **Latent Factor** | **Indicator** | **B** | **SE** | **Z** | **Beta** | **sig** |
| --- | --- | --- | --- | --- | --- | --- |
| amotivation | W1_M1_5 | 1.000 | 0.000 |  | 0.733 |  |
| amotivation | W1_M2_4 | 1.002 | 0.073 | 13.672 | 0.644 | *** |
| amotivation | W1_M3_1 | 0.969 | 0.067 | 14.456 | 0.705 | *** |
| amotivation | W1_M3_6 | 1.203 | 0.095 | 12.694 | 0.586 | *** |
| external | W1_M1_4 | 1.000 | 0.000 |  | 0.618 |  |
| external | W1_M2_3 | 0.919 | 0.069 | 13.321 | 0.602 | *** |
| external | W1_M3_3 | 1.157 | 0.084 | 13.777 | 0.628 | *** |
| social | W1_M3_8 | 1.000 | 0.000 |  | 0.463 |  |
| social | W1_M2_7 | 1.035 | 0.095 | 10.834 | 0.403 | *** |
| introjected | W1_M1_7 | 1.000 | 0.000 |  | 0.634 |  |
| introjected | W1_M2_2 | 1.341 | 0.090 | 14.916 | 0.730 | *** |
| introjected | W1_M2_8 | 1.383 | 0.090 | 15.413 | 0.769 | *** |
| introjected | W1_M3_7 | 1.261 | 0.089 | 14.205 | 0.681 | *** |
| identified | W1_M1_3 | 1.000 | 0.000 |  | 0.511 |  |
| identified | W1_M1_8 | 1.137 | 0.098 | 11.554 | 0.553 | *** |
| identified | W1_M3_4 | 1.154 | 0.095 | 12.083 | 0.594 | *** |
| integrated | W1_M1_2 | 1.000 | 0.000 |  | 0.657 |  |
| integrated | W1_M2_1 | 1.207 | 0.081 | 14.866 | 0.681 | *** |
| integrated | W1_M2_5 | 1.152 | 0.079 | 14.659 | 0.669 | *** |
| integrated | W1_M3_5 | 1.311 | 0.081 | 16.160 | 0.760 | *** |
| intrinsic | W1_M1_1 | 1.000 | 0.000 |  | 0.551 |  |
| intrinsic | W1_M1_6 | 1.273 | 0.102 | 12.476 | 0.667 | *** |
| intrinsic | W1_M2_6 | 1.392 | 0.104 | 13.449 | 0.767 | *** |
| intrinsic | W1_M3_2 | 1.258 | 0.097 | 12.983 | 0.715 | *** |
| *Note*: *p<0.05; **p<0.01; *** p<0.001 | | | | | | |

**Table 1b.** Factor loadings of model with social motivation factor

Moreover, the different types of motivation were moderately to strongly correlated and did not approximate the SDT predicted simplex pattern (where scores representing motivational subscales that are closer together on the proposed self-determination continuum are more strongly and positively correlated than scores derived from subscales intended to represent constructs that are expected to be more distal; see Table 2). As an example, the latent variable intrinsic motivation was correlated to both extrinsic forms of motivation (e.g., external regulation) and intrinsic forms of motivation (e.g., integrated regulation).

|  | Amotivated | External | Introjected | Identified | Integrated | Intrinsic | Social |
| --- | --- | --- | --- | --- | --- | --- | --- |
| Amotivated regulation | (0.32 ^***^) | **0.31** ^***^ | **0.10** | **0.08** | **-0.04** | **-0.02** | **0.30** ^***^ |
| External regulation | *0.14* ^***^ | (0.80 ^***^) | **0.52** ^***^ | **0.83** ^***^ | **0.67** ^***^ | **0.77** ^***^ | **0.73** ^***^ |
| Introjected regulation | *0.05* | *0.39* ^***^ | (0.95 ^***^) | **0.72** ^***^ | **0.86** ^***^ | **0.55** ^***^ | **0.45** ^***^ |
| Identified regulation | *0.04* | *0.74* ^***^ | *0.61* ^***^ | (0.80) | **0.86** ^***^ | **0.94** ^***^ | **0.78** ^***^ |
| Integrated regulation | *-0.03* | *0.57* ^***^ | *0.79* ^***^ | *0.79* ^***^ | (1.09 ^***^) | **0.75** ^***^ | **0.58** ^***^ |
| Intrinsic regulation | *-0.02* | *0.63* ^***^ | *0.46* ^***^ | *0.90* ^***^ | *0.69* ^***^ | (1.02 ^***^) | **0.66 ^***^** |
| Social motivation | *0.14* ^***^ | *0.83* ^***^ | *0.32* ^***^ | *0.57* ^***^ | *0.42* ^***^ | 0.53 ^***^ | (0.38 ^***^) |
| *Notes*: Terms below the diagonal represent covariances (in italics); diagonal terms represent variances (in parentheses); terms above the diagonal represent Pearson correlation coefficients (in bold).  *p<0.05; **p<0.01; *** p<0.001 | | | | | | |  |

**Table 2.** (Co)variance matrix for the SMS-6 model with the added social motivation factor

**References**

Epskamp, S. (2015). semPlot: Unified Visualizations of Structural Equation Models. *Structural Equation Modeling: A Multidisciplinary Journal*, *22*(3), 474–483. https://doi.org/10.1080/10705511.2014.937847

Merkle, E. C., & You, D. (2018). *Getting started with nonnest2*. Available online at: https://cran.r-project.org/web/packages/nonnest2/vignettes/nonnest2.pdf (accessed June 2, 2021).

Merkle, E. C., You, D., & Preacher, K. J. (2016). Testing nonnested structural equation models. *Psychological Methods*, *21*(2), 151.

Vuong, Q. H. (1989). Likelihood Ratio Tests for Model Selection and Non-Nested Hypotheses. *Econometrica*, *57*(2), 307–333. https://doi.org/10.2307/1912557
