# Supplementary material for: Running Together: How Sports Partners Keep You Running
Source: Front Sports Act Living. 2022 Mar 16;4:643150. doi: 10.3389/fspor.2022.643150 (PMC8966768; doi:10.3389/fspor.2022.643150)
Supplement: Supplementary Datasheet 3 — Appendix B. [file Data_Sheet_3.docx]

# Appendix B: preliminary analyses

## Relation between running frequency and show up

We described the development of the mean running frequency of the people who eventually attended the race and of those who did not show up (see Figure 1). We can observe that the non-attenders ran significantly less frequently in the weeks before the race than the people who went on to attend. However, months after the race, the difference between the attenders and non-attenders is non-significant, suggesting that the non-attenders caught up again. We tentatively interpret these data as follows: people gave up on the goal of participating in the Seven Hills Run (e.g., because they set too ambitious a goal), which resulted in decreased training frequency, but only temporarily. Given the strong relation between training frequency and attendance, a logistic regression on attendance forms a strong robustness check for our analyses of running frequency. After all, this yields greater statistical power (higher sample size, no between-within design). However, even though these two indicators are highly interrelated, they do not seem to be two sides of the same coin, because a lot of non-attenders continued to train. Therefore, it remains theoretically relevant to explore how networks affect running frequency and attendance rates differently.


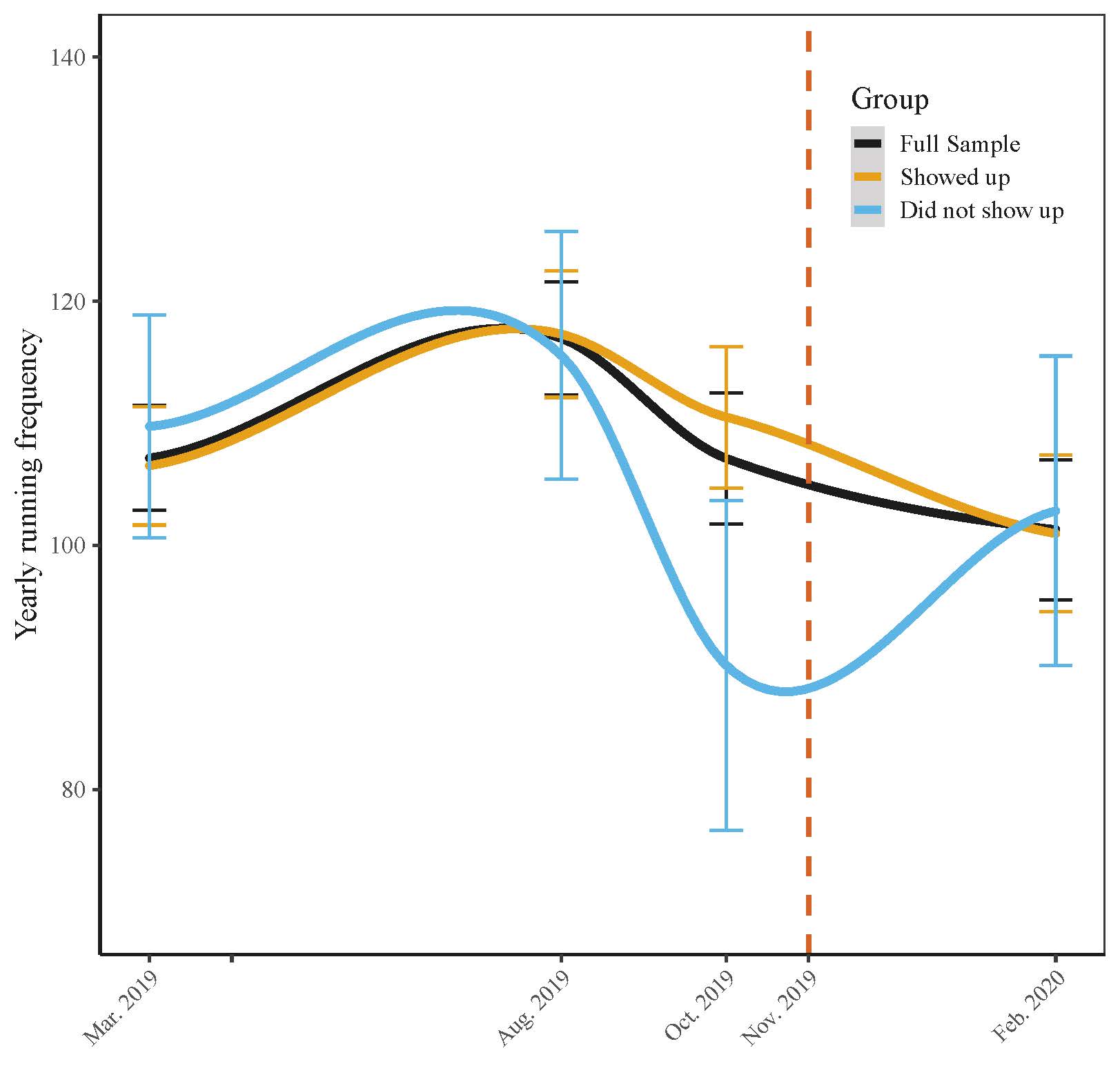

**Figure 1.** Development of running frequency for attenders and non-attenders.
*Notes*: Horizontal lines depict error bars representing the standard error of the mean. Orange vertical dotted line represents the Seven Hills Run. For each timepoint, we included all observations with valid scores on running frequency.

## Composition of the CSN

One of the name interpreter questions put for each sport partner referred to their relationship with the respondents. These data were used to explore how the CSN is formed and who it is made up of. Figure 2 shows the categories respondents could choose to describe their relationship with their alters, and their corresponding prominence in the CSN. Overall, it seems that the CSN is very diverse, but that respondents were most likely to report friends as important co-runners, closely followed by their partner or fellow club members.

**
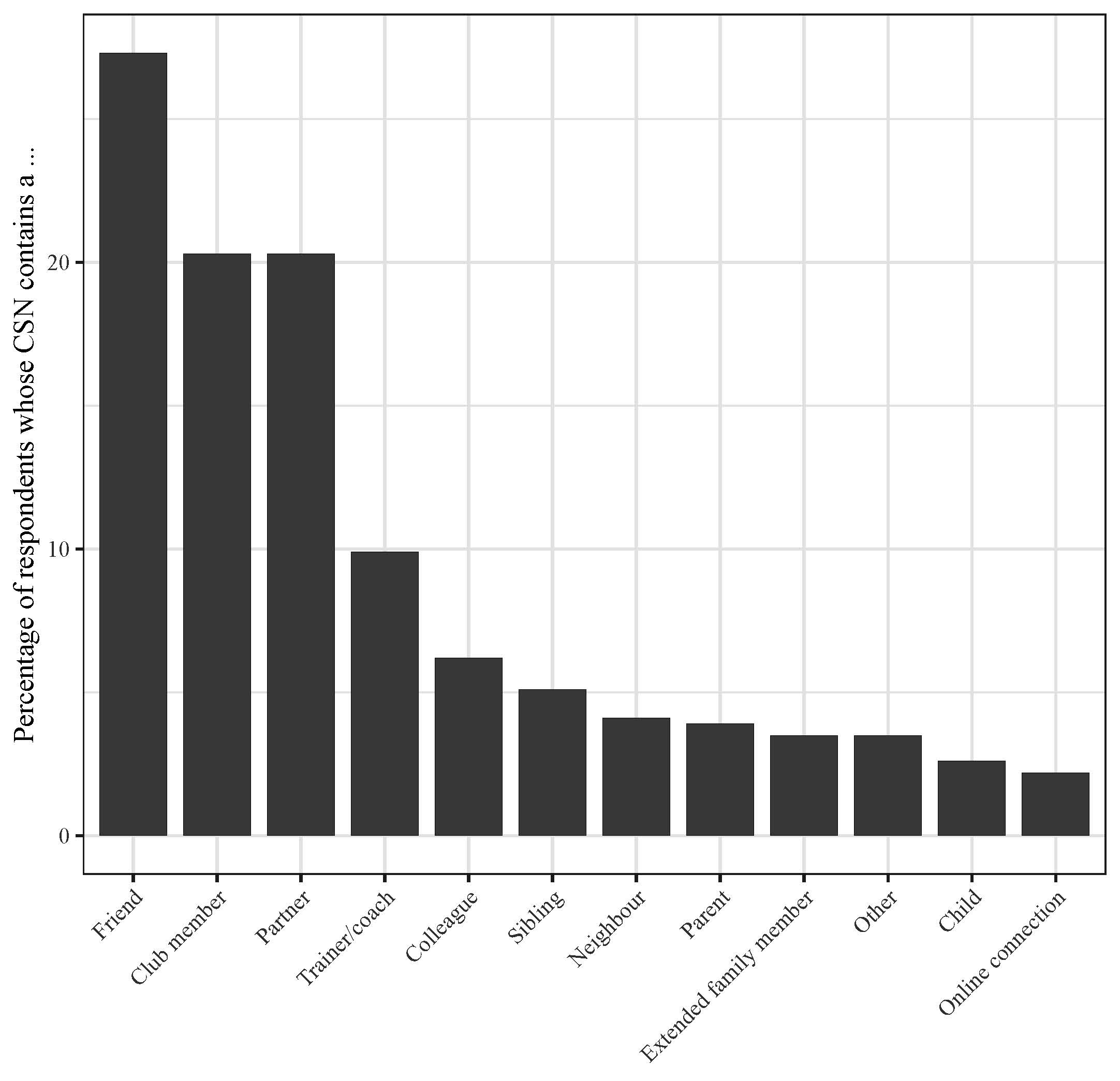
Figure 2.** Whom does the Core Sports Network consist of?
*Notes*: Bars reflect the percentage of respondents (N=802) whose CSN included at least one of the respective relationships (at Wave 1). X-axis labels refer to the categories respondents were given to describe the relationship with their alters (multiple options possible); Y-axis refers to the percentage of CSNs including an (at least one) alter who was described as having the particular role.
