# Supplementary material for: Running Together: How Sports Partners Keep You Running
Source: Front Sports Act Living. 2022 Mar 16;4:643150. doi: 10.3389/fspor.2022.643150 (PMC8966768; doi:10.3389/fspor.2022.643150)
Supplement: Supplementary Datasheet 4 — Appendix C. [file Data_Sheet_4.docx]

# Appendix C: robustness checks

## Different network measures

As a first robustness check, we re-estimated our between-within models with separate variables for the number of more, equally and less competent co-runners. Results are presented in Table 1.
 **Table 1.** Between-within effects of number of more/equally/less competent co-runners

|  | **Model 1** | | **Model 2** | |
| --- | --- | --- | --- | --- |
|  | *b* | *SE* | *b* | *SE* |
| (Intercept) | 110.88 ^***^ | 17.72 | 110.90 ^***^ | 17.73 |
| ***Within-effects*** |  |  |  |  |
| More competent co-runners | 6.90 | 4.24 | 6.15 | 4.38 |
| Equally competent co-runners | -6.65 | 4.12 | -6.66 | 4.23 |
| Less competent co-runners | 7.91 ^~^ | 4.64 | 6.64 | 4.84 |
| ***Between-effects*** |  |  |  |  |
| More competent co-runners | -4.40 | 4.35 | -4.33 | 4.36 |
| Equally competent co-runners | 0.52 | 4.66 | 0.58 | 4.66 |
| Less competent co-runners | 7.17 | 5.23 | 7.23 | 5.24 |
| Sports club | 25.04 ^***^ | 5.54 | 25.13 ^***^ | 5.55 |
| Commercial gym | 15.03 ^*^ | 7.65 | 14.97 | 7.65 |
| Informal group | 5.54 | 6.17 | 5.58 | 6.18 |
| Online sport network | 7.91 | 5.25 | 7.82 | 5.26 |
| Social motivation | 2.94 | 2.26 | 2.71 | 2.27 |
| ***Cross-level interaction*** |  |  |  |  |
| Higher * social motivation |  |  | 2.64 | 4.21 |
| Equal * social motivation |  |  | 1.51 | 3.71 |
| Lower * social motivation |  |  | 3.28 | 4.31 |
| Individuals (n) / Observations (N) | 642 / 1117 | | 642 / 1117 | |
| Marginal R^2^ / Conditional R^2^ | 0.119 / 0.565 | | 0.119 / 0.565 | |
| *Notes*: ~ p<0.1; * p<0.05; ** p<0.01; *** p<0.001 (two-tailed) Only explanatory variables of interest are shown, excluding controls and time fixed-effects. Also, only fixed intercepts ($\beta_{0}$) and slopes ($\beta_{1}^{'}$) are shown, and not the random parts ($u_{0i}$ and $u_{1i}$). No within-effects of sport setting (e.g., sports club) are estimated, as this variable was invariant between waves 1 and 2. All coefficients not shown are available upon request. | | | | |

Second, we replaced our network measures with the number of “strong ties” and “weak ties”. Table 2 presents the results for running frequency; Table 3 for show up. In our logistic model, we also performed mediation analysis by including both (X) the number of co-runners that are (not) more competent, and (M) the number of strong and weak ties (Table 3, Model 2). The results indicate mediation: the relationship between X and Y was eliminated when M is controlled.

**Table 2.** Between-within effects of number of strong and weak tie co-runners, separately

|  | **Model 1** | | **Model 2** | |
| --- | --- | --- | --- | --- |
|  | *b* | *SE* | *b* | *SE* |
| (Intercept) | 110.82 ^***^ | 17.70 | 111.17 ^***^ | 17.67 |
| ***Within-effects*** |  |  |  |  |
| Strong tie co-runners | -1.28 | 2.98 | -0.01 | 3.19 |
| Weak tie co-runners | -3.40 | 2.78 | -3.50 | 2.83 |
| ***Between-effects*** |  |  |  |  |
| Strong tie co-runners | 0.73 | 4.44 | 0.77 | 4.45 |
| Weak tie co-runners | -2.71 | 4.87 | -2.93 | 4.86 |
| Sports club | 24.69 ^***^ | 5.49 | 24.78 ^***^ | 5.49 |
| Commercial gym | 15.04 | 7.65 | 15.36 | 7.64 |
| Informal group | 4.80 | 6.11 | 4.22 | 6.09 |
| Online sport network | 7.86 | 5.24 | 7.72 | 5.24 |
| Social motivation | 3.39 | 2.27 | 3.24 | 2.28 |
| ***Cross-level interaction*** |  |  |  |  |
| Strong * social motivation |  |  | -5.91 ^~^ | 3.04 |
| Weak * social motivation |  |  | -1.16 | 2.51 |
| Individuals (n) / Observations (N) | 642 / 1117 | | 642 / 1117 | |
| Marginal R^2^ / Conditional R^2^ | 0.113 / 0.559 | | 0.114 / 0.586 | |
| *Notes*: ~ p<0.1; * p<0.05; ** p<0.01; *** p<0.001 (two-tailed) Only explanatory variables of interest are shown, excluding controls and time fixed-effects. Also, only fixed intercepts ($\beta_{0}$) and slopes ($\beta_{1}^{'}$) are shown, and not the random parts ($u_{0i}$ and $u_{1i}$). No within-effects of sport setting (e.g., sports club) are estimated, as this variable was invariant between waves 1 and 2. All coefficients not shown are available upon request. | | | | |

**Table 3.** Logistic regression effects on showing up of alter competence and tie strength

|  | **Model 1** | | **Model 2** | |
| --- | --- | --- | --- | --- |
|  | *OR* | *CI* | *OR* | *CI* |
| (Intercept) | 1.33 | 0.28 – 6.73 | 1.37 | 0.28 – 6.96 |
| More competent co-runners |  |  | 1.32 | 0.50 – 3.49 |
| Equally/less competent co-runners |  |  | 1.89 | 0.71 – 5.06 |
| Strong ties | 1.51 ^*^ | 1.06 – 2.19 | 0.92 | 0.33 – 2.61 |
| Weak ties | 1.48 ^~^ | 0.97 – 2.38 | 0.97 | 0.38 – 2.58 |
| Sports club | 0.85 | 0.49 – 1.48 | 0.87 | 0.51 – 1.53 |
| Commercial gym | 0.75 | 0.37 – 1.64 | 0.76 | 0.37 – 1.67 |
| Informal group | 1.47 | 0.78 – 2.94 | 1.50 | 0.79 – 3.02 |
| Online sport network | 0.91 | 0.54 – 1.49 | 0.93 | 0.55 – 1.53 |
| Social motivation | 1.13 | 0.90 – 1.42 | 1.12 | 0.90 – 1.42 |
| Observations | 649 | | 649 | |
| R^2^ Tjur | 0.045 | | 0.048 | |
| *Notes*: * p<0.05; ** p<0.01; *** p<0.001 (two-tailed). The dependent variable show up was coded so that 0 = did not show up and 1 = did show up. OR = odds ratio; CI = 95 % confidence interval; significances are based on the log(odds).  Only explanatory variables of interest are shown, excluding controls. All coefficients not shown are available upon request | | | | |

## Different operationalizations of motivation

We re-estimated our between-within model by controlling for the standardized weighted sum scores of the other subdimensions of the SMS-6. Results are presented in Table 4.

We also explored motivational profiles in our sample of runners using Latent Profile Analysis (LPA) and SDT as our theoretical framework. As a first step, we scanned our SMS-6 data for string responding (>10) and multivariate outliers with Mahalanobis Distance (*α*<0.10), using the R-package ‘careless’ (Yentes & Wilhelm, 2018). We performed CFA and calculated weighted sum scores for each of the original SDT subscales. Subsequently, we performed LPA using the R-package ‘Mclust’ (Scrucca et al., 2016) with as input variables the sum scores. We estimated a sequence of increasingly complex models (by incrementally increasing the number of profiles). We sought to choose the most meaningful model following SDT while also considering model fit, guided by recommendations in the literature (Nylund et al., 2007) and previous research (e.g., Gustafsson et al. 2018). We identified three motivational profiles (see Figure 1); these profiles resemble the previously identified motivational profiles among elite level athletes by Gustafsson et al. (2018): (1) an *amotivated* *and moderately controlled* (henceforth: *amotivated*) profile, with athletes displaying very high levels of amotivation, slightly above average levels of extrinsic forms of motivation (i.e. extrinsic and introjected regulation), and average scores on intrinsic forms of motivation (identified, integrated and intrinsic regulation); (2) a *low motivation* profile with athletes expressing low levels of both extrinsic and intrinsic forms of motivation, and low amotivation; (3) a *high motivation* profile with athletes displaying high levels of intrinsic motivation and moderate to high levels of extrinsic forms of motivation, but low amotivation. Note that our typology of motivational profiles is based on standardized z-scores, meaning that they reflect standard deviation (SD) units above or below the sample mean (which is set to 0). Average posterior probabilities support the three-class solution, with high probabilities that athletes belong to their assigned profiles (average posterior probabilities for all profiles above 0.89).

**
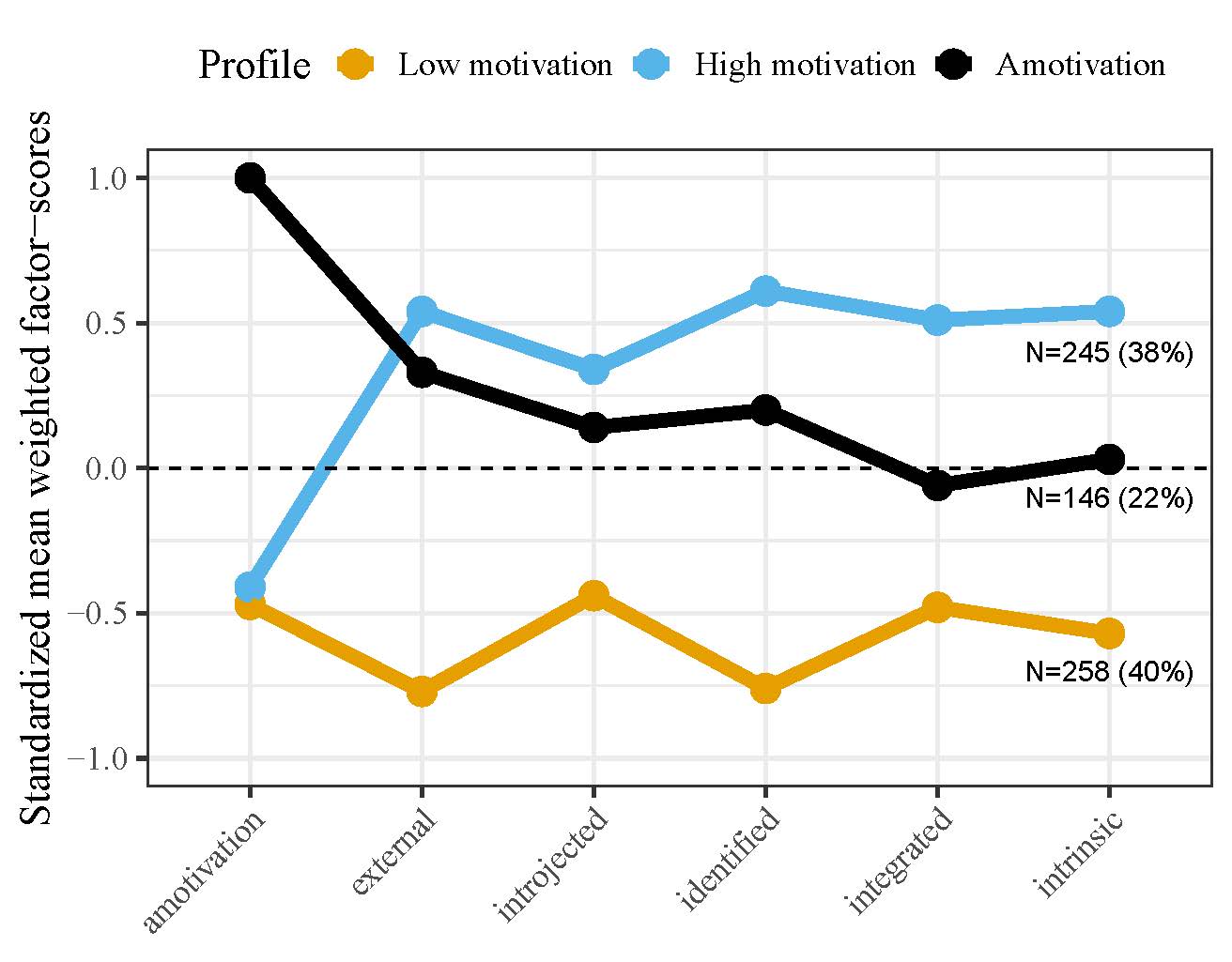

Figure 1.** Description of latent motivational profiles based on SDT
*Notes*: Dashed horizontal line reflects sample means (set to 0). Dots depict standardized mean weighted factor scores for three motivational profiles (i.e. SD units above/below the mean). Values exceeding +1 SD were trimmed.

We re-estimated our models but now instead of including our social motivation measure, we included our motivational profiles as dummies (with the amotivated profile as the reference category). Results are presented in Table 4.

|  | ***Motivation control variables*** | | | | ***Motivational profiles (LPA)*** | | | | |
| --- | --- | --- | --- | --- | --- | --- | --- | --- | --- |
|  | **Model 1** | | **Model 2** | | **Model 3** | | **Model 4** | | |
|  | *b* | *SE* | *b* | *SE* | *b* | *SE* | *b* | *SE* |  |
| (Intercept) | 81.99 ^***^ | 15.68 | 82.49 ^**^ | 15.69 | 82.05 ^***^ | 15.90 | 82.53 ^***^ | 15.94 |  |
| ***Within-effects*** |  |  |  |  |  |  |  |  |  |
| CSN size | 4.02 ^~^ | 2.15 | 3.43 | 2.64 | 4.03 ^~^ | 2.15 | 3.98 | 5.80 |  |
| Sports club | 15.68 ^*^ | 6.90 | 18.39 ^**^ | 7.14 | 15.48 ^*^ | 6.90 | 18.30 ^*^ | 7.14 |  |
| Commercial gym | 7.12 | 7.34 | 7.47 | 7.50 | 6.99 | 7.34 | 7.54 | 7.50 |  |
| Informal group | -1.66 | 5.14 | -2.82 | 5.15 | -1.70 | 5.14 | -2.70 | 5.14 |  |
| ***Between-effects*** |  |  |  |  |  |  |  |  |  |
| CSN size | -1.10 | 3.29 | -1.03 | 3.28 | 0.26 | 3.29 | 0.27 | 3.29 |  |
| Sports club | 27.66 ^***^ | 5.14 | 27.71 ^***^ | 5.15 | 29.70 ^***^ | 5.12 | 29.64 ^***^ | 5.13 |  |
| Commercial gym | 16.86 ^*^ | 7.60 | 17.15 ^*^ | 7.63 | 18.72 ^*^ | 7.70 | 19.04 ^*^ | 7.73 |  |
| Informal group | 8.92 | 6.13 | 9.27 | 6.18 | 7.19 | 6.15 | 7.57 | 6.21 |  |
| Online sport network | 5.24 | 4.60 | 5.10 | 4.61 | 6.11 | 4.67 | 6.00 | 4.68 |  |
| Social motivation | -0.90 | 2.70 | -1.12 | 2.72 |  |  |  |  |  |
| Amotivation | -3.66 | 2.59 | -3.60 | 2.59 |  |  |  |  |  |
| External | 1.78 | 2.78 | 1.91 | 2.79 |  |  |  |  |  |
| Introjected | -3.04 | 2.36 | -2.96 | 2.36 |  |  |  |  |  |
| Identified | 4.66 ^~^ | 2.74 | 4.59 ^~^ | 2.74 |  |  |  |  |  |
| Integrated | 4.66 ^*^ | 2.74 | 5.38 ^*^ | 2.65 |  |  |  |  |  |
| Intrinsic | 3.90 | 2.5 | 3.88 | 2.56 |  |  |  |  |  |
| Low motivations (ref.=amotivated) |  |  |  |  | 2.81 | 4.86 | 2.69 | 4.91 |  |
| High motivations (ref.=amotivated) |  |  |  |  | 13.24 ^**^ | 4.95 | 13.15 ^**^ | 5.00 |  |
| ***Cross-level interaction*** |  |  |  |  |  |  |  |  |  |
| CSN size * social motivation |  |  | 1.13 | 2.42 |  |  |  |  |  |
| CSN size * low motivations |  |  |  |  |  |  | 0.21 | 6.93 |  |
| CSN size * high motivations |  |  |  |  |  |  | -1.24 | 6.99 |  |
| Individuals (n) / Observations (N) | 647 /2229 | | 647 / 2229 | | 647 /2229 | | 647 / 2229 | | |
| Marginal R^2^ / Conditional R^2^ | 0.139 / 0.570 | | 0.140 / 0.607 | | 0.125 / 0.570 | | 0.125 / 0.607 | | |
| Notes: ~ p<0.1; * p<0.05; ** p<0.01; *** p<0.001 (two-tailed) Only explanatory variables of interest are shown, excluding controls and time fixed-effects. Also, only fixed intercepts ($\beta_{0}$) and slopes ($\beta_{1}^{'}$) are shown, and not the random parts ($u_{0i}$ and $u_{1i}$). | | | | | | | | | |

**Table 4.** Between-within effects of CSN size with other operationalizations of motivation

**References**

Gustafsson, H., Carlin, M., Podlog, L., Stenling, A., & Lindwall, M. (2018). Motivational profiles and burnout in elite athletes: A person-centered approach. *Psychology of Sport and Exercise*, *35*, 118–125. https://doi.org/10.1016/j.psychsport.2017.11.009

Nylund, K. L., Asparouhov, T., & Muthén, B. O. (2007). Deciding on the Number of Classes in Latent Class Analysis and Growth Mixture Modeling: A Monte Carlo Simulation Study. *Structural Equation Modeling: A Multidisciplinary Journal*, *14*(4), 535–569. https://doi.org/10.1080/10705510701575396

Scrucca, L., Fop, M., Murphy, T. B., & Raftery, A. E. (2016). mclust 5: Clustering, Classification and Density Estimation Using Gaussian Finite Mixture Models. *The R Journal*, *8*(1), 289–317.

Yentes, R. D., & Wilhelm, F. (2018). careless: Procedures for computing indices of careless responding. R package version 1.1.0. Available at: https://github.com/ryentes/careless (accessed June 2, 2021).
